# Genomic discovery of an evolutionarily programmed modality for small-molecule targeting of an intractable protein surface

**DOI:** 10.1073/pnas.2006560117

**Published:** 2020-06-30

**Authors:** Uddhav K. Shigdel, Seung-Joo Lee, Mathew E. Sowa, Brian R. Bowman, Keith Robison, Minyun Zhou, Khian Hong Pua, Dylan T. Stiles, Joshua A. V. Blodgett, Daniel W. Udwary, Andrew T. Rajczewski, Alan S. Mann, Siavash Mostafavi, Tara Hardy, Sukrat Arya, Zhigang Weng, Michelle Stewart, Kyle Kenyon, Jay P. Morgenstern, Ende Pan, Daniel C. Gray, Roy M. Pollock, Andrew M. Fry, Richard D. Klausner, Sharon A. Townson, Gregory L. Verdine

**Affiliations:** ^a^Warp Drive Bio, Inc., Redwood City, CA 94063;; ^b^Department of Molecular and Cell Biology, University of Leicester, LE1 7RH Leicester, United Kingdom;; ^c^Lyell Immunopharma, South San Francisco, CA 94080;; ^d^Department of Stem Cell and Regenerative Biology, Harvard University, Cambridge, MA 02138;; ^e^Department of Chemistry and Chemical Biology, Harvard University, Cambridge, MA 02138;; ^f^Department of Molecular and Cellular Biology, Harvard University, Cambridge, MA 02138

**Keywords:** natural products, genome mining, FK506-binding protein

## Abstract

The vast majority of intracellular protein targets are refractory toward small-molecule therapeutic engagement, and additional therapeutic modalities are needed to overcome this deficiency. Here, the identification and characterization of a natural product, WDB002, reveals a therapeutic modality that dramatically expands the currently accepted limits of druggability. WDB002, in complex with the FK506-binding protein (FKBP12), potently and selectively binds the human centrosomal protein 250 (CEP250), resulting in disruption of CEP250 function in cells. The recognition mode is unprecedented in that the targeted domain of CEP250 is a coiled coil and is topologically featureless, embodying both a structural motif and surface topology previously considered on the extreme limits of “undruggability” for an intracellular target. Structural studies reveal extensive protein–WDB002 and protein–protein contacts, with the latter being distinct from those seen in FKBP12 ternary complexes formed by FK506 and rapamycin. Outward-facing structural changes in a bound small molecule can thus reprogram FKBP12 to engage diverse, otherwise “undruggable” targets. The flat-targeting modality demonstrated here has the potential to expand the druggable target range of small-molecule therapeutics. As CEP250 was recently found to be an interaction partner with the Nsp13 protein of the SARS-CoV-2 virus that causes COVID-19 disease, it is possible that WDB002 or an analog may exert useful antiviral activity through its ability to form high-affinity ternary complexes containing CEP250 and FKBP12.

## Significance

This manuscript reports on a member of the FK506/rapamycin family, WDB002, and the realization that FKBP-mediated recognition is a genetically programmable modality that enables engagement of topologically flat targets. FKBP-mediated recognition is thus nature’s strategy for drugging the “undruggable.” The surface of FKBP engages three completely unrelated targets—calcineurin, MTOR, and CEP250—with high-target affinity and specificity, using different constellations of amino acid residues. Target specificity is determined solely by the “variable domain” of the bound small molecule alone, suggesting the modality might be generalizable to other undruggable targets through variable domain engineering. Finally, since WDB002 targets CEP250, it may be a promising starting point for developing a treatment for COVID-19.

Approximately 3,000 proteins encoded in the human genome are predicted to bind drug-like small molecules and are thus considered druggable (1, 2). To date, only a small fraction of these proteins has been linked to human disease and represent viable therapeutic targets (3); given this limited overlap, new intervention points and or technologies for therapeutic targeting are badly needed. Protein–protein interactions (PPIs), which are important to most cellular processes and not accounted for in these estimates of druggability, represent potential intervention points (4). Notwithstanding that, protein–protein interfaces have proven difficult to target (5) because the protein surfaces involved are topologically flat and do not provide the pockets or crevices well established as being necessary for small-molecule binding. Although protein therapeutics expand the number of potential therapeutic targets, they do not penetrate cell membranes (5), so do not address PPIs inside the cell (4). Thus, modalities that access intracellular PPIs and other intracellular proteins refractory to small molecules and biologics would expand the repertoire of viable therapeutic target opportunities.

**Fig. 1.**
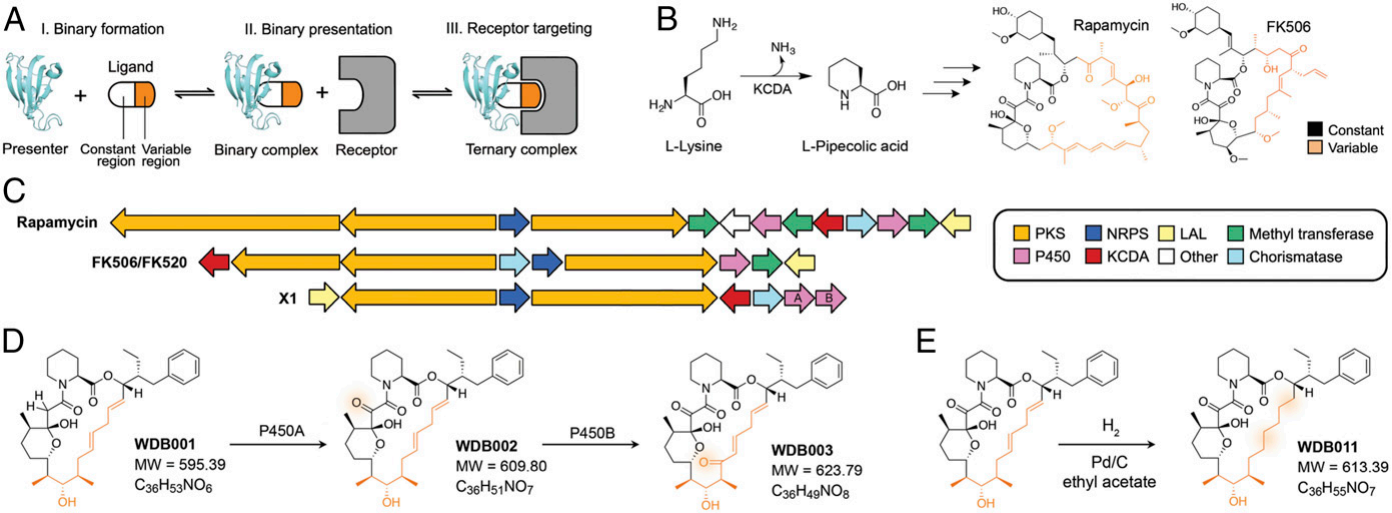
Discovery of the FKBP12-binding compounds WDB001, -002, and -003 and synthesis of WDB011, a semisynthetic analog. (*A*) Schematic of the presenter-based chemical modality. A presenter protein forms a binary complex with the small molecule via the constant region (white) of that molecule. In the context of the binary complex, the composite presenter protein–small-molecule interface, comprising the variable region of the small molecule (orange) and amino acids from the presenter protein, mediates ternary complex formation with the receptor. (*B*) KCDA catalyzes the formation of a key rapamycin and FK506 precursor. Constant and variable regions are shown in black and orange, respectively. (*C*) Comparative BGC analysis of rapamycin, FK506, and X1. (*D*) X1-encoded compounds WDB001 to WDB003 resulting from successive oxidations by BGC P450 genes (colored as rapamycin and FK506 in *B*. Structural variations from WDB002 are indicated by orange shading. (*E*) WDB011, a semisynthetic analog generated by chemical reduction of WDB002.

Natural products have proven a valuable source of new therapeutics, with ∼50% of FDA-approved small-molecule drugs being ultimately derived from biological sources (6). Forged by the relentless forces of survival and evolution, these natural molecules often achieve their biologic effects by accessing mechanisms unprecedented with anthropogenic drugs, enabled in part by structural complexity far exceeding that of typical synthetic drugs. A prominent example of such evolutionarily derived mechanistic innovation is found with rapamycin and FK506, structurally related hybrid NRPS/PKS natural products derived from *Streptomycetes* (Fig. 1*B*) (7–10). Rapamycin and FK506 derive their clinically useful immunosuppressive activity from their binding and inhibiting mTOR and calcineurin, respectively. However, neither rapamycin nor FK506 alone can stably bind their target (11, 12); instead, both are “presented” to their target as a tightly bound complex with a ubiquitous, abundant cellular protein, the peptidyl prolyl isomerase FK506-binding protein (FKBP12) (13–15). FK506 and rapamycin contain two structural elements, a “constant region” that plunges deeply into FKBP12 and confers high-affinity binding (Fig. 1 *A* and *B*, black), and a “variable region” that is displayed on the surface of FKBP12 (Fig. 1 *A* and *B*, orange). Interestingly, though FKBP12 makes extensive protein–protein interactions with both mTOR and calcineurin upon ternary complex formation, the variable region in rapamycin and FK506 alone is responsible for programming target selectivity. In both instances, the hydrocarbon-rich variable region of the drug provides a hydrophobic “hotspot” (16) absent in FKBP12 alone, which engages a chemically complementary hotspot on the target; such hotspots are a hallmark and often essential feature of protein–protein interactions (17, 18). The relatedness of the biosynthetic gene clusters (BGCs) encoding FK506 and rapamycin suggested they arise from a common ancestor, which to us further suggested these might be the founding examples of a genetically programmable modality deployed more broadly in nature to engage intractable targets.

As a rigorous and expansive test of this hypothesis, we interrogated the genomes of ∼135,000 *Actinomycete* species to identify new members of the FK506/rapamycin structural class. We report the characterization of X1, a BGC from *Streptomyces malaysiensis* DSM41697, whose products comprise a family of FK506- and rapamycin-like, cell-permeable small molecules. We show that a subset of compounds encoded by X1, in complex with FKBP12, bind to a topologically flat surface within the coiled-coil domain of the human centrosomal protein CEP250 (also known as C-Nap1). This work shows that complexation with FKBP12 enables a small molecule to accomplish what was previously considered impossible—to bind a completely flat recognition surface. Furthermore, this work reports a small molecule engaging and modulating the activity of a coiled coil, a structural domain previously thought “undruggable” by small molecules. The present findings definitively establish that the surface of FKBP12 is capable of forming cooperative ternary complexes with multiple targets having completely different folds and surface functionality, and therefore we suggest that FKBP12-assisted targeting via ternary complex formation should be considered a broadly enabling modality that holds promise to drug proteins currently beyond therapeutic reach.

## Results

### X1 Encodes FK506/Rapamycin-Like Products.

To discover BGCs encoding members of the FK506/rapamycin structural class (FK gene clusters), we developed a biosynthetic “search term” to query our microbial sequence database containing partial sequences of pooled DNA samples from ∼135,000 *Actinomycete* species. We searched for BGCs harboring the lysine cyclodeaminase (KCDA) gene, which encodes the enzyme that catalyzes the conversion of lysine to pipecolate (Fig. 1*B*) (19). Pipecolate is incorporated in the final and shared step of FK506 and rapamycin assembly-line biosynthesis (19, 20) and directly binds the FKBP active site. Once we identified genetic matches to the search term in our fragmentary sequence database, we identified the candidate *Actinomycete* strains harboring putative FK gene clusters. Our genome-mining strategy initiates by searching for a single gene biosynthetic hallmark of an FK gene cluster, the lysine cyclodeaminase gene, with a second round of deep sequencing and assembly to reveal the entire cluster (Fig. 1*C*). Via this genome-mining strategy, we rediscovered clusters encoding the known family members FK506/FK520, rapamycin, and antascomycin (21); we also discovered seven clusters (X1, X11, X22, X23, X15, X35, and X36) encoding natural products with constant region genes closely related to those of that FK506, rapamycin, and antascomycin, but with variable regions that bore substantial coding differences from previously known family members and from each other. Five of these clusters were cloned, overexpressed, and demonstrated through extensive structural characterization to encode variants of FK506 and rapamycin (*SI Appendix*, Table S1) having reprogrammed variable-region structures. Below we present in-depth characterization of the most prevalent cluster, X1, and its products.

We identified the X1 FK cluster from *S. malaysiensis* DSM41697 through genome mining as described above, polished the cluster through exhaustive whole-genome sequencing, and characterized its products WDB001–WDB003 by engineered overexpression, fermentation, isolation, and structure elucidation (Fig. 1 *C* and *D* and *SI Appendix*, Fig. S1). WDB001 is the primary product of X1; it can undergo two P450-mediated oxidation reactions that install a carbonyl group at C9, yielding WDB002, and at C18, yielding WDB003 (Fig. 1*D*). We also report WDB011, a semisynthetic, stabilized analog of WDB002 with the diene reduced (Fig. 1*E*). Similar to rapamycin and FK506, each product contains a conserved pipecolate-containing structural element that we designate as a constant region (Fig. 1 *B* and *D*, black), responsible for interaction with FKBP12, as well as a variable region, that in FK506 and rapamycin engage the therapeutic target, and in the X1 products, has a smaller polyketide chain length than the variable region of rapamycin and FK506 (Fig. 1 *B* and *D* orange). The C22–23 double bond has an atypical placement, resulting from δ-elimination rather than the stereotypical β-elimination during polyketide chain elaboration (*SI Appendix*, Fig. S1 *A* and *C*). Unlike rapamycin and FK506, the exocyclic starter unit is aromatized (Fig. 1 *B* and *D* and *SI Appendix*, Fig. S1). We predicted that the structural differences in the variable region and starter unit chemistry of WDB001–WDB003 and WDB0011 would “reprogram” it to interact with a different protein target than rapamycin and FK506. With the exception of WDB001, which is missing a key carbonyl, the core of the constant regions of these compounds matches that of rapamycin.

### X1-Encoded Compounds Bind Human CEP250.

**Fig. 2.**
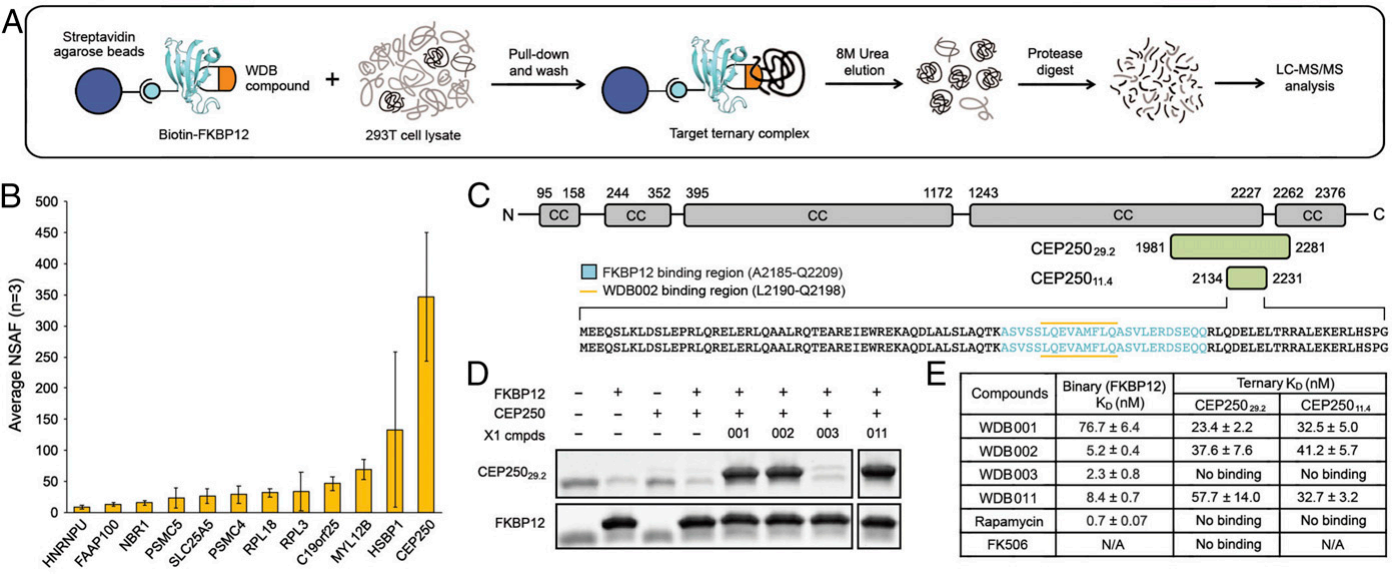
CEP250 is the primary target of FKBP12–WDB002. (*A*) Target-ID CEP250 showed a significant total spectral count (NSAF average = 347) proteomics workflow to identify the protein targets of FKBP12–WDB002. (*B*) CEP250 showed a total spectral count (NSAF average = 347) for WDB002 samples (*n* = 3) with no CEP250 peptides observed in DMSO control experiments (*n* = 3). Error bars are SD of three independent experiments. (*C*) Schematic of CEP250, showing the full-length protein and minimal interacting regions, CEP250_29.2_ and CEP250_11.4_, located in the C-terminal domain region. For more detail on the identification of the minimal interaction regions, see *SI Appendix*, Fig. S2 and *SI Methods*. (*D*) Pull-down experiments show CEP250_29.2_ binds WDB001, WDB002, and WDB011 but not WDB003 in the presence of FKBP12 in vitro. (*E*) Summary table of binary and ternary K_D_ measurements by SPR for WDB001, WDB002, WDB003, WDB011, rapamycin, and FK506. For sensorgrams and full kinetic data, see *SI Appendix*, Figs. S3 and S4 and Table S2.

In order to characterize the target of the FKBP12–WDB002 complex, we developed an affinity-based proteomic protocol in HEK293T cell lysates exogenously supplied with biotinylated FKBP12 and either dimethyl sulfoxide (DMSO) or 10 µM WDB002 (Fig. 2*A*). To identify candidate targets for the FKBP12–WDB002 binary complex, we analyzed DMSO- and WDB002-containing samples; these experiments reproducibly revealed the presence of CEP250 only in the WDB002-treated samples with an average normalized spectral abundance factor (NSAF) of 347 (Fig. 2*B*). CEP250 was not identified in DMSO- or FK506-treated lysates. These data suggest that CEP250, a 250-kDa centrosome-associated protein, is a primary target for WDB002.

To confirm CEP250 is a direct target of the X1-encoded compounds and to explore the molecular interactions mediated by these compounds, we set out to identify a minimal domain of CEP250 required for FKBP12-natural product binding (Fig. 2*C*). Because the variable regions of WDB001 and WDB002 are identical, we utilized WDB001 for the domain mapping experiments. We performed in vitro pull-down experiments using recombinantly expressed domains from CEP250 including the N-terminal domain (1 to 362), two intermediate domains (I1: 380 to 1,200 and I2: 1,219 to 2,124), and the C-terminal domain (1,982 to 2,442) (*SI Appendix*, Fig. S2*A*). FKBP12–WDB001 engaged the C-terminal domain only (*SI Appendix*, Fig. S2*B*), demonstrating that this region is the site for ternary complex formation and supporting the FKBP12–WDB002–CEP250 interaction observed in the proteomic experiments. By expressing a series of C-terminal domain truncations (R0–R11; *SI Appendix*, Fig. S2*C*) via in vitro transcription and translation, we identified a 29.2-kDa minimal-binding region composed of residues 1,981 to 2,281 (CEP250_29.2_; *SI Appendix*, Fig. S2*D*) interacting with FKBP12–WDB001.

We next assessed whether the X1-encoded compounds WDB001 to 003 and WDB011 in combination with FKBP12 bind CEP250 in vitro. We performed pull-down experiments using recombinant CEP250_29.2_ in the presence of biotinylated-FKBP12 and the X1-encoded compounds. Using streptavidin magnetic beads to harvest the complexes, we found that CEP250_29.2_ binds WDB001, WDB002, and WDB011 in vitro; we did not detect interaction between FKBP12–WDB003 and CEP250_29.2_ (Fig. 2*D*). Together, these data indicate that WDB001, WDB002, and WDB011 interact with FKBP12 to bind CEP250, whereas WDB003 does not. Given the lack of evidence supporting an interaction between FKBP12–WDB003 and CEP250, we omitted WDB003 from further analyses and it is not analyzed further here.

All compounds formed binary complexes with FKBP12 with nanomolar affinity. Because WDB001 is missing a key carbonyl, we predicted this compound might bind FKBP12 with lower affinity than the other X1-encoded compounds; indeed, it binds 15-fold weaker than WDB002 (Fig. 2*E*). WDB011, a synthetic derivative of WDB002 with the diene reduced, binds FKBP12 with similar affinity as WDB002 (Fig. 2*E* and *SI Appendix*, Table S2). In complex with FKBP12, WDB001, WDB002, and WDB011 each bind CEP250_29.2_ with nanomolar affinity (Fig. 2*E* and *SI Appendix*, Table S2). No measurable interaction with CEP250_29.2_ was detected for any compound alone without FKBP12 (*SI Appendix*, Fig. S3 and Table S2). These findings are consistent with the explicit dependence on FKBP12 binding for rapamycin and FK506 to engage mTOR and calcineurin (17, 18). The synthetic compound, WDB011, retained the potency of the natural compounds for CEP250.

In addition to CEP250, 11 other targets were identified in the proteomic target-identification (ID) screen of FKBP12–WDB002, albeit less reproducibly and with significantly lower spectral counts than CEP250 (Fig. 2*B*), suggesting that they either bind weakly to FKBP12:WDB002, or they are associated with the complex containing CEP250. To determine the specificity of FKBP12–WDB002 for CEP250 we tested the direct binding of FKBP12–WDB002 to heat shock factor-binding protein 1 (HSBP1) (22), a 76-amino acid coiled-coil protein that was identified as having the next highest spectral counts after CEP250. No measurable interaction with recombinant HSBP1 was detected for FKBP12–WDB002 (*SI Appendix*, Fig. S5 and Table S2), providing further support that CEP250 is the primary target of WDB002.

### FKBP12–WDB002 Binds a Coiled Coil.

**Fig. 3.**
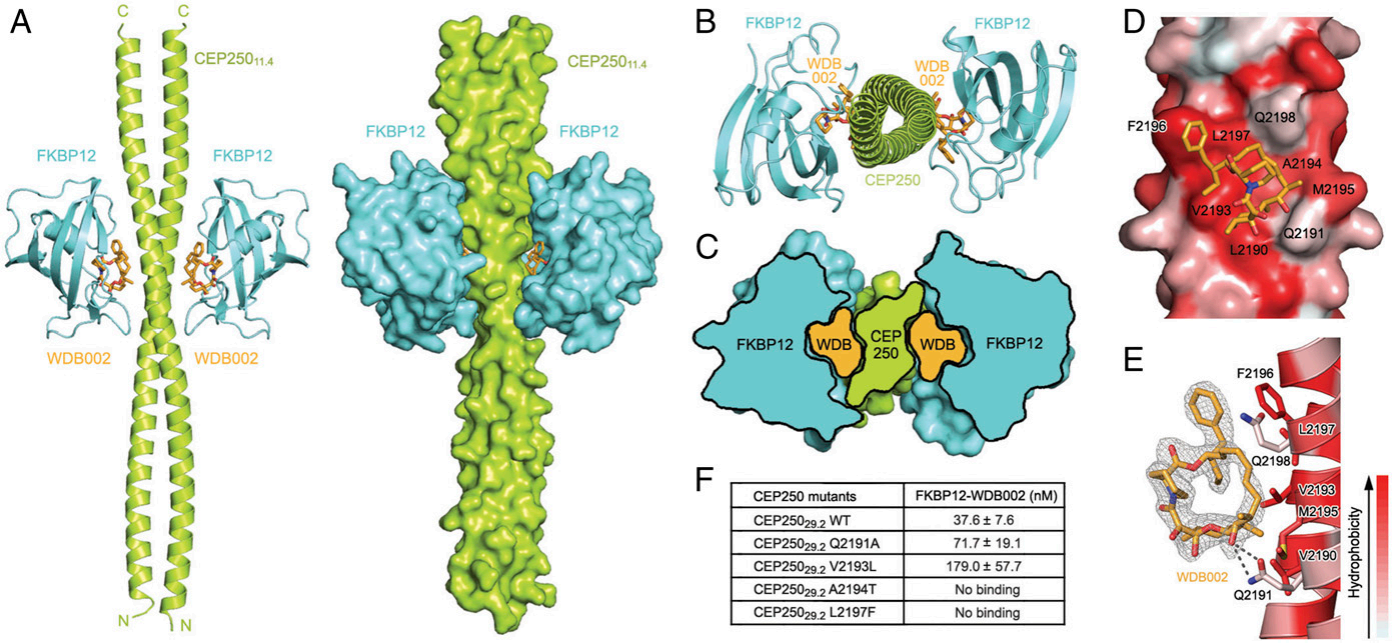
Crystal structure of the FKBP12–WDB002–CEP250 ternary complex. (*A*) Overall structure of the FKBP12 (cyan) –WDB002 (orange sticks)–CEP250_11.4_ (green) ternary complex shown as a cartoon (*Left*) and with surface representation (*Right*). Carboxyl (C) and amino (N) termini of CEP250_11.4_ are labeled. (*B*) Top-down view of the ternary complex showing the architecture of the CEP250 coiled coil. (*C*) Cross-sectional surface representation view of the ternary complex highlighting penetration of WDB002 into both FKBP12 and CEP250. (*D*) WDB002 (orange sticks) binds a hydrophobic hotspot on a flat surface of the CEP250_11.4_ coiled coil. The degree of CEP250_11.4_ surface hydrophobicity is displayed by a color gradient (red: most hydrophobic; white: least hydrophobic). (*E*) Schematic showing the CEP250_11.4_ side chains (sticks, colored as in *D*) involved in WDB002 binding. Hydrogen bonds are shown as dotted lines, and f_o_–f_c_ density contoured at 3.0 σ for WDB002 is shown in gray mesh. A π–π interaction occurs between the phenyl headgroup of WDB002 and Phe2196. We omit FKBP12 from *D* and *E* for clarity. (*F*) Summary table of SPR ternary K_D_ measurements for FKBP12–WDB002 binding to hotspot mutations on CEP250. For sensorgrams and full kinetic data, see *SI Appendix*, Fig. S7 and Table S2.

CEP250 is a core component of the centrosome and is predominantly composed of sequence predicted to form a coiled coil (23). The discovery of a series of compounds that bind to a coiled coil is unexpected as the flat surface of these protein interfaces provides none of the features typically considered necessary for small-molecule binding (5). To explore how complexes of FKBP12 and X1-encoded compound bind CEP250, we employed X-ray crystallography. During crystallization, CEP250_29.2_ underwent spontaneous proteolysis, yielding a degradation product of CEP250 consisting of residues 2,134 to 2,231 (11.4 kDa, hereafter referred to as CEP250_11.4_), which was sufficient to bind X1-encoded compounds in vitro with comparable affinity (Fig. 2*E* and *SI Appendix*, Fig. S4 and Table S2). We report the structure of CEP250_11.4_ bound to FKBP12 in the presence of WDB002 (Fig. 3, Protein Data Bank [PDB] ID 6OQA). FKBP12–WDB002–CEP250_11.4_ crystallizes as a heterotetramer with C2 symmetry, in which CEP250_11.4_ forms a homodimeric coiled coil and binds one FKBP12–WDB002 complex on each side of the coiled coil (Fig. 3 *A*–*D*).

Similar to rapamycin and FK506 (17, 18), WDB002 is embedded in the FKBP12-target protein interface, making extensive interaction with both FKBP12 and CEP250_11.4_ (Fig. 3 *A*–*C* and *SI Appendix*, Fig. S6). The WDB002 constant region binds in a hydrophobic pocket of FKBP12, which is formed by highly conserved aromatic residues (*SI Appendix*, Fig. S8). Comparison with the ternary structures of rapamycin and FK506 shows that the core of the WDB002 constant region makes identical key hydrogen bonding contacts with FKBP12 as do rapamycin and FK506, consistent with the observation that this region is shared between all three compounds (*SI Appendix*, Fig. S8). Differences exist, however, in how WDB002, rapamycin, and FK506 interact with FKBP12 outside of the catalytic pocket, including residue-specific van der Waals contacts to the variable region and starter unit. The WDB002 structure has the smallest variable region and starter unit, as compared with rapamycin and FK506 (Fig. 1 *A* and *D*), and correspondingly makes the fewest number of contacts with FKBP12 outside of the catalytic pocket, which may contribute to its fivefold weaker binding affinity to FKBP12 (Fig. 2*E*).

The two FKBP12–WDB002 binary complexes contact CEP250 independently; both interact with the CEP250 dimer, contacting both strands of the coiled coil. Consistent with the rapamycin and FK506 ternary structures, FKBP12–WDB002 binds CEP250 via a composite interface, with FKBP12 binding to the constant region of WDB002 and presenting the variable region to CEP250 (Fig. 3 *A*–*C* and *SI Appendix*, Fig. S6). The overall buried surface area (BSA) between WDB002–FKBP12 and CEP250 is ∼1,700 Å^2^ and is comparable to typical PPI interfaces (1,500 to 3,000 Å^2^) (24, 25).

The variable region of WDB002 buries an extremely flat, hydrophobic patch of the CEP250_11.4_ dimer interface composed of Leu2190, Val2193, Phe2196, and Leu2197 from one protomer and Gln2191, Ala2194, Met2195, and Gln2198 from the other. Nonhydrophobic contacts between FKBP12 and CEP250 also appear critical for binding, as evidenced by the number of interprotein hydrogen bonds. Side chains of Gln2191, Gln2198, and Gln2209 from one CEP250_11.4_ protomer form hydrogen bonds with the side chain of Asp37, and the main chain carbonyl groups of Lys47 and Gly19 of FKBP12. Arg2204 and Ser2189 from the other CEP250_11.4_ protomer form hydrogen bonds with the main-chain carbonyl groups of Lys52 and Pro88 of FKBP12, respectively (*SI Appendix*, Fig. S6). This abundance of interprotein hydrogen bonds was not observed in previously reported FKBP12 ternary complexes (17, 18) and is large for a PPI (26).

The CEP250 dimer interacts with WDB002 via a hydrophobic hotspot (16, 27) composed of Leu2190, Val2193, Phe2196, and Leu2197 from one protomer and Gln2191, Ala2194, Met2195, and Gln2198 from the other. As expected for a coiled coil, this surface is devoid of any prominent grooves or pockets that are typically necessary for small-molecule binding. Despite this flat architecture, WDB002 binds this hydrophobic hotspot (Fig. 3 *D* and *E*) in complex with FKBP12. Similar to rapamycin and FK506, the variable region of WDB002 provides the complementary hydrophobic hotspot with a BSA of ∼510 Å^2^ between WDB002 and CEP250.

To confirm the direct interaction of the CEP250 hydrophobic hotspot with WDB002, we introduced single-point hotspot mutations onto CEP250_29.2_ and determined binding affinities to FKBP12–WDB002 (Fig. 3*F*). Two mutations, Gln2191Ala and Val2193Leu, attenuated the binding affinity by twofold and fivefold, respectively, whereas binding was completely abolished with the introduction of the single mutations Ala2194Thr and Leu2197Phe (*SI Appendix*, Table S2 and Fig. S7), underscoring the importance of these hotspot residues in WDB002 recognition and binding. Furthermore, the extra carbonyl possessed by WDB003 at the C18 position is predicted to sterically clash with the main chains of hotspot residues Ala2194 and Met2195 and is consistent with the lack of any detected interaction with CEP250 (Fig. 3*D*).

Two key interactions occur at the WDB002–CEP250 interface (Fig. 3*E*). The C16 hydroxyl group, the only hydrophilic functional group presented to CEP250, forms two hydrogen bonds with the side-chain carbonyl and amine groups of Gln2191 from one CEP250_11.4_ protomer (Fig. 3*E*). At the opposite end of the structure, the aromatic starter unit of WDB002 makes a π–π interaction with Phe2196 from the other protomer (Fig. 3*E*). All other atoms presented to CEP250 from WDB002 interact via van der Waals interactions at a flat hydrophobic surface. This structure provides evidence that the FKBP12 and X1-encoded compounds together form a composite interface that drives high-affinity binding to a CEP250 hotspot, despite the flat, seemingly undruggable surface of the coiled coil.

### Plasticity of FKBP12 Facilitates Binding to Multiple Targets.

**Fig. 4.**
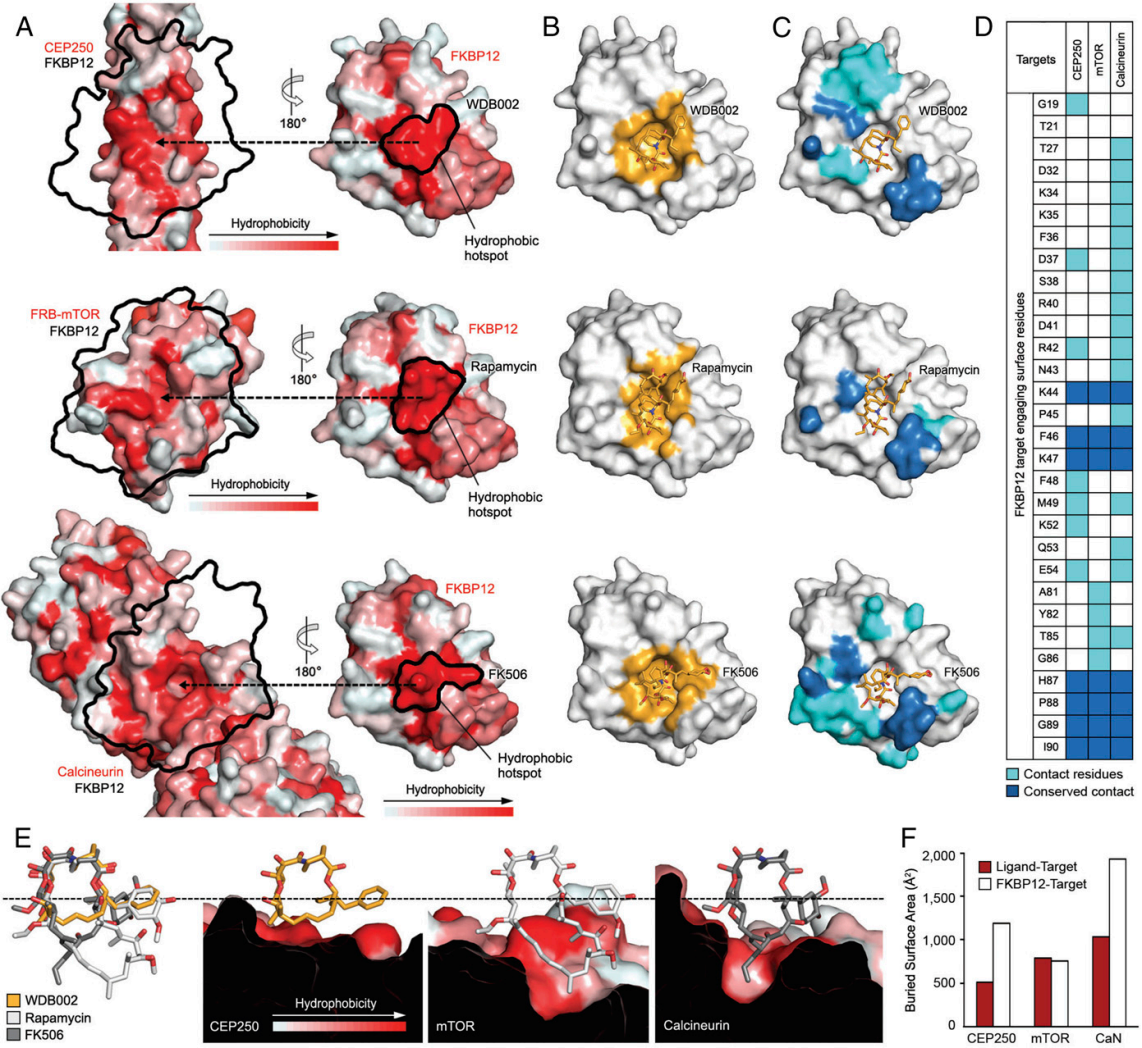
Plasticity of FKBP12 facilitates binding to multiple targets. (*A*) Hydrophobic hotspots on the target proteins, CEP250_11.4_ coiled-coil dimer (*Upper Left*), FRB domain of mTOR (*Middle Left*), and calcineurin (*Lower Left*) interact with hotspots created at the FKBP12–small-molecule interface (WDB002 [*Upper Right*], rapamycin [*Middle Right*], and FK506 [*Lower Right*]). Hydrophobicity is displayed as in Fig. 3. Black arrows and black outlines indicate the small-molecule binding sites and the position of FKBP12 on the targets, respectively. (*B*) FKBP12 binds the three small molecules with overlapping but distinct amino acids (yellow). (*C*) FKBP12 deploys different residues to interact with each target protein. Contact residues for each complex are shown (cyan) as well as the seven residues that are utilized in all complexes (blue). (*D*) Graphic illustration of FKBP12 residues deployed in each complex. (*E*) Superposition of WDB002 (yellow), rapamycin (white), and FK506 (gray) (*Left*), and side view of the three compounds interacting with their respective targets. WDB002 uses a flat hydrophobic surface to bind the flat hydrophobic surface of the CEP250 coiled coil. Rapamycin projects a conjugated triene arm and methyl groups into a hydrophobic crevice of mTOR’s FKBP12–rapamycin-binding (FRB) domain. FK506 lodges an allyl group into a large hydrophobic cleft on human calcineurin. Portions of the structure above the dotted line comprise the “constant region” of the natural product; below, “variable region.” (*F*) BSA formed between FKBP12 and the target (white) or the compound and the target (red) in the CEP250, mTOR, and calcineurin (CaN) complexes.

X1-encoded compounds share FKBP12 as a presenter protein with rapamycin and FK506. Clam-shell views of three ternary structures (Fig. 4*A*; FKBP12–WDB002–CEP250, FKBP12–rapamycin–mTOR, and FKBP12–FK506–calcineurin) illustrate that the interfaces between the target proteins and the FKBP12-compound complexes are all formed by contiguous patches of hydrophobic residues, or hotspots (16, 27). Importantly, the extent to which the small molecules and FKBP12 contribute to target engagement on the presenter–small-molecule side of the complex differs dramatically, demonstrating the adaptability of the presenter modality.

This plasticity is enabled by FKBP12, a chaperone protein that has evolved to bind diverse protein substrates to catalyze cis-trans proline isomerization (28, 29). FKBP12 binds the small molecules similarly via mainly conserved contacts (Fig. 4*B*) but utilizes a different repertoire of residues to engage each of the three target proteins (Fig. 4 *C* and *D*). Indeed, only seven residues from FKBP12 overlap across the three protein–protein interfaces, with many of these residing in the 80s loop (Ala84–Ile91), a flexible loop that lies adjacent to the catalytic pocket (30) (Fig. 4 *C* and *D* and *SI Appendix*, Fig. S9). However, even these seven shared contact residues are deployed differently: for example, FKBP12 uses the side chain of Phe46 to contact CEP250 but uses the side chain and main chain of this residue in the other complexes (*SI Appendix*, Fig. S6). The variable deployment results from both side-chain rotation and also in part from changes to the backbone structure of FKBP12 that alter its end-to-end length by 2.5 Å across the complexes, with the most dramatic residue displacements (>2.0 Å) occurring in the 80s loop (*SI Appendix*, Fig. S9). This loop is reported to undergo target-mediated conformational changes upon binding calcineurin and mTOR, forming key interactions with both targets (17, 18). In the CEP250 structure the 80s loop extends away from the target interface, as compared with calcineurin and mTOR, most likely to accommodate binding to a flat surface (*SI Appendix*, Fig. S9). In this way, the loop acts as a flexible recognition motif that can adapt to binding multiple targets.

The adaptability of the modality can also be seen in the relative contributions of FKBP12 or the small molecule to the interfaces (Fig. 4*F*). In the FKBP12–rapamycin–mTOR complex, in addition to providing a hydrophobic hotspot (Fig. 4*A*), rapamycin provides half of the BSA (790 of 1,548 Å^2^) (Fig. 4*F*). Rapamycin also projects a conjugated triene arm and methyl groups into a hydrophobic crevice of mTOR’s FKBP12–rapamycin-binding (FRB) domain (Fig. 4*E*). The fact that FKBP12 can be replaced by multiple FK506-binding proteins in this complex, which differ notably from FKBP12 both in the identity and location of residues deployed (31, 32) (*SI Appendix*, Fig. S10), suggests that rapamycin makes stronger ligand–target interactions than FKBP12 and is responsible for a larger fraction of the overall interaction energy driving formation of this complex.

In the FKBP12–WDB002–CEP250 complex, FKBP12 primarily facilitates complex formation, with WDB002 providing a hydrophobic hotspot (Fig. 4*A*) but only ∼30% of the BSA (Fig. 4*F*; 510 of 1,700 Å^2^). The variable region of the X1-encoded compounds distinguishes these complexes from those formed by FK506 and rapamycin. All X1-encoded compounds have a small macrocyclic ring, which interacts with CEP250; this ring is two and nine carbon atoms shorter than that of FK506 and rapamycin, respectively (Fig. 1 *B* and *D*). Unlike the cyclohexyl moiety in rapamycin and FK506, the aryl starter unit makes direct contact with the target. In this ternary complex, FKBP12 forms a large number of hydrogen bonds with CEP250 across the interface and provides the bulk of the BSA (1,190 Å^2^). FKBP12.6, which differs from FKBP12 by only three amino acids at the interface with CEP250 (*SI Appendix*, Fig. S10; D37N, M49R, and I90V), cannot replace FKBP12, supporting the notion that FKBP12-target interactions primarily promote formation of this ternary complex.

### WDB002 Disrupts NEK2-Mediated Centrosome Separation.

Next, we set out to determine whether WDB002 can modulate the biological activity of CEP250 in human cells. CEP250 is a component of the centrosome, the primary microtubule-organizing center of mammalian cells. Each cell has two centrosomes that are held in close proximity during interphase by a proteinaceous linker. CEP250 is a component of this filamentous structure, specifically connecting it to the centrosomes (33). At the onset of mitosis, NEK2 phosphorylates CEP250 and triggers its displacement from the centrosome (23, 34–37). This leads to disassembly of the linker, in a process called centrosome disjunction, which precedes centrosome separation and bipolar spindle assembly in mitosis (38). Interfering with linker disassembly alters the timing of centrosome separation promoting chromosome segregation errors in mitosis (39, 40). Moreover, genetic depletion of CEP250 increases the distance between centrosomes (from 1 to 2.5 µm) in interphase cells disrupting Golgi organization and cell migration (41).

**Fig. 5.**
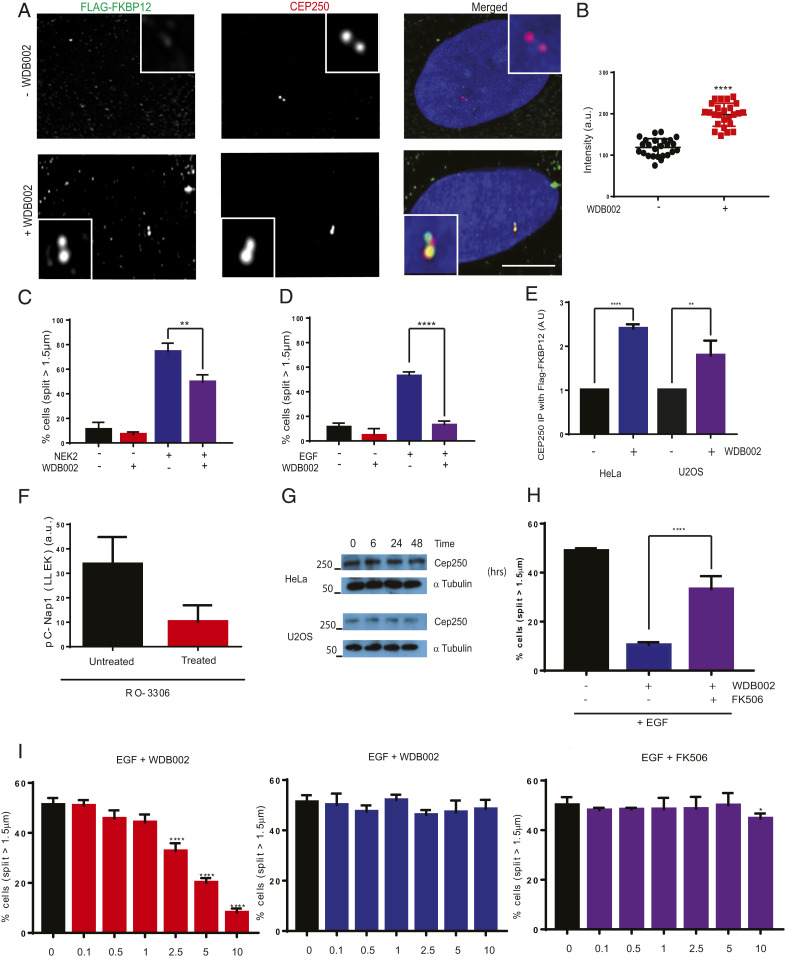
WDB002 recruits FKBP12 to the centrosome and blocks NEK2- and EGF-induced centrosome separation. (*A*) U2OS:Flag-FKBP12 cells were treated with or without WDB002 for 24 h before being fixed and stained with antibodies against Flag (green) and CEP250 (red); DNA was stained with Hoechst 33258 (blue). (Scale bar, 5 µm.) The overlapping but incomplete colocalization of Flag-FKBP12 and CEP250 is consistent with FKBP12 binding the C-terminal domain of CEP250 and the antibody against CEP250 being raised against its N-terminal domain. (*B*) The dot plot indicates intensity of Flag staining at the centrosome in the presence and absence of WDB002. (*C*) Histogram shows the percentage of cells with centrosomes split by >1.5 µm following treatment with or without WDB002 and NEK2 as indicated. (*D*) Histogram shows the percentage of cells with centrosomes split by >1.5 µm following treatment with or without WDB002 and EGF as indicated. (*E*) Histogram shows the percentage of cells with centrosomes split by >1.5 µm following treatment with EGF together with no drug, WDB002, or FK506 as indicated. (*F*) Histograms showing percentage of cells with centrosomes split by >1.5 µm following treatment with EGF together with increasing doses (µM) of WDB002, WDB003, or FK506 as indicated. (*G*) Histogram showing intensity measurements of centrosome stained with phospho-CEP250 antibodies in the presence or absence of WDB002. (*H*) Western blot analysis of CEP250 and α-tubulin expression in HeLa and U2OS cell lysates following incubation with WDB002 for the times indicated. Molecular weights (kDa) are indicated. (*I*) Histograms indicate the amount of myc-CEP250 immunoprecipitated with FLAG-FKBP12 from lysates of HeLa and U2OS cells incubated in the presence, relative to the absence, of WDB002. *P < 0.05, **P < 0.01, ****P < 0.0001.

To determine whether WDB002 can direct ternary complex formation of CEP250 with FKBP12 in cells and impact on centrosome organization and regulation, we evaluated its activity in U2OS cells stably expressing FKBP12 bearing an amino-terminal FLAG tag. We first examined the localization of FKBP12 and CEP250 by immunofluorescence microscopy with antibodies to the FLAG epitope and endogenous CEP250. In the absence of WDB002, when FKBP12 and CEP250 do not associate, FLAG-FKBP12 was distributed throughout the cytoplasm, whereas CEP250 localized to the centrosome (Fig. 5*A*). Treatment of U2OS cells with 10 μM of WDB002 for 24 h resulted in colocalization of FKBP12 with CEP250 at the centrosome, consistent with WDB002 promoting interaction of FKBP12 with CEP250 in cells. Quantification of FLAG staining at centrosomes defined by CEP250 staining demonstrated a significant WDB002-dependent increase in the association of FKBP12 with this structure (Fig. 5*B*).

To determine whether WDB002 affects centrosome organization, we quantified the impact of WDB002 on the distance between the two centrosomes in asynchronous interphase U2OS cells. Treatment with 10 µM WDB002 moderately decreased the percentage of cells with centrosomes separated by a distance greater than 1.5 μm (Fig. 5 *C* and *D*). However, the effect of WDB002 treatment was more pronounced when centrosome separation was stimulated by overexpression of wild-type NEK2 (Fig. 5*C*) or incubation with EGF (Fig. 5*D*), treatments known to trigger premature centrosome separation through CEP250 phosphorylation (37, 39). Indeed, the inhibition of EGF-induced centrosome splitting by WDB002 could be reversed by coincubation with FK506, consistent with these two compounds competing for FKBP12 (Fig. 5*E*). However, while EGF-induced separation was dose dependent for WDB002 (with significant inhibition observed at 2.5 µM), there was little or no inhibition with either WDB003 or FK506 (Fig. 5*F*). The ability of WDB002 to interfere with NEK2-dependent phosphorylation of CEP250 was demonstrated by reduced centrosome staining with an antibody directed against CEP250-pSer2064 (37) (Fig. 5*G*). This was not a result of WDB002-induced degradation of CEP250 as the total abundance of CEP250 was unchanged by this compound (Fig. 5*H*). Finally, we found that coprecipitation of FLAG-FKBP12 with a myc-tagged C-terminal domain construct of CEP250 encoding residues 1,964 to 2,442 was significantly increased in the presence as compared to absence of WDB002 (Fig. 5*I*).

Together, these data support a model whereby WDB002 recruits FKBP12 to CEP250 at the centrosome and impacts centrosome linker organization, resulting in decreased centrosome separation. Moreover, WDB002-mediated recruitment of FKBP12 to CEP250 appears to interfere specifically with the ability of NEK2 to actively stimulate centrosome disjunction through CEP250 phosphorylation. We note that the WDB002–FKBP12 binding site (residues 2,185 to 2,209) is close to, but not overlapping, the NEK2–CEP250 interaction region (residues 2,362 to 2,442), while directly overlying a number of NEK2 phosphorylation sites in CEP250 (36). Further studies will be required to gain a detailed mechanistic understanding of the impact of WDB002 on centrosome disjunction and how this affects centrosome function.

## Conclusion

Here we have reported the use of a genome-mining approach to discover seven biosynthetic gene clusters encoding an entire ensemble of diverse members of the rapamycin/FK506 family of hybrid NRPS/PKS natural products. The physical organization of these biosynthetic gene clusters strongly suggests an evolutionary relationship with the clusters encoding rapamycin and FK506, where homologous recombination within the portion of the PKS encoding the variable domain has reprogrammed the chemical structure of the variable domain. The changes in the variable domain are both necessary and sufficient to reprogram the target specificity of these molecules. The products of the X1 cluster have an additional element of structural novelty, an aryl starter unit in place of the dihydroxycyclohexyl found in FK506 and rapamycin; notwithstanding the difference, the different starter units share a common origin in shikimate.

Our study further reveals that the surface of FKBP is remarkably versatile, utilizing different residues and surface chemistry to enable the formation of high-affinity, high-specificity cooperative complexes with three completely different target proteins. In these complexes, the polyketide stripe of the variable domain provides the hydrophobic hotspot that is key for productive protein–protein interactions but lacking in FKBP. Thus, the bound natural product completes the surface of FKBP and also programs its surface chemistry to engage a chemically complementary surface on the target. It stands to reason that the surface of FKBP could be deployed in yet other ways to engage additional targets, and that alterations of the variable region via combinatorial biosynthesis, semisynthesis, or de novo synthesis could reprogram the binary FKBP–small-molecule complex to engage novel targets. We suggest that FKBP-assisted target recognition should be viewed as a broadly enabling targeting modality. The fungal natural product cyclosporin A engages a second prolyl isomerase, cyclophilin, to form a binary complex that targets the same region of calcineurin as FK506/FKBP (11). Though reprogrammed variants of cyclosporine are not known, the current observations suggest that they exist, and furthermore that cyclophilin-dependent recognition of intractable targets could be engineered in much the same way as FKBP.

Whereas the surfaces of mTOR and calcineurin targeted by rapamycin and FK506, respectively, show some degree of invagination, the surface of CEP250 contacted by WDB002 is essentially flat. Not only has such a flat surface never before been shown capable of binding a small molecule, but also never before has a coiled coil been shown targetable at high affinity by a small molecule. In a recent cellular protein interactome screen for SARS-CoV-2, the causative agent of COVID-19 disease, CEP250 was identified as a host factor that is engaged by the viral Nsp13 protein (42), suggesting the possibility that WDB002 may carry inhibitory activity against SARS-CoV-2 by binding to CEP250. The present results provoke a thorough reevaluation and expansion of protein targets that should be considered druggable and suggest that FKBP-assisted targeting can enable even the flattest of protein targets to be productively engaged by orally active small molecules.

## Materials and Methods

DNA for ∼135,000 actinomycete strains were obtained from partner organizations, representing legacy pharmaceutical collections or publicly available strains, and sequenced by WuXi Genomics (Shanghai, China) using Illumina HiSeq 2×100 chemistry. Strains that contain a large PKS cluster next to the KCDA resulted in acquisition of the strain, growth, and isolation of high molecular weight DNA for whole-genome sequencing, including PacBio SMRT sequencing. Dried crude extract from large-scale pellets expressing X1 cluster were fractioned by medium-pressure liquid chromatography , followed by reversed phase high-performance liquid chromatography to purify WDB001, WDB002, and WDB003. Human FKBP12, CEP250_11.4_, CEP250_29.2_, and CEP250_29.2_ mutants containing various tags were expressed in *Escherichia coli* BL21(DE3)pLysS cells using standard protocols. To identify the binding target of X1-encoded compounds, N-terminal AVI-tagged and biotinylated FKBP12 was added to the HEK293T lysate to a final concentration of 4 µM and supplemented with 10 µM compound and streptavidin agarose at 4 °C for 60 min. Eluted proteins were digested and analyzed using liquid chromatography with tandem mass spectrometry (LC-MS/MS) on a Velos-Pro OrbiTrap mass spectrometer operated in data-dependent Top20 mode with collision-induced dissociation-based fragmentation. We identified the peptides using Sequest with a target-decoy database. For crystallization, CEP250_11.4_ was incubated with three-molar excess of FKBP12 and nine-molar excess of WDB002 at 4 °C overnight. FKBP12–WDB002–CEP250 ternary complex was isolated by size exclusion chromatography and then subjected to crystallization screening. Diffraction quality crystals were obtained under the condition of 0.1 M Hepes 7.0; 0.2 M sodium malonate; 21% PEG 3350 by the sitting-drop vapor diffusion method. Data collection and structure determination were carried out using standard procedures. Binary and ternary-binding kinetics were measured at 25 °C on a Biacore S200 surface plasmon resonance (SPR) instrument. To assess binding kinetics of FKBP12-binding ligands, AVI-FKBP12 was immobilized on an NTA sensor chip followed by injecting the compounds over a concentration range. Ternary K_D_ was measured using a similar protocol except that AVI-FKBP12 was saturated with compound in the buffer and CEP250 was injected over a concentration range. The effects of WDB002 on FKBP12 and CEP250 colocalization and centrosome separation were assessed in U2OS cells bearing a stably integrated murine stem cell virus retroviral vector expressing FKBP12 with an amino terminal FLAG epitope. Cells were incubated with WDB002 and FK506 at 10 µM unless otherwise indicated. Immunofluorescence microscopy was performed with primary antibodies to the FLAG epitope (Sigma F3165) and CEP250 (Cambridge Bioscience 14498) and images were captured using a Leica TCS SP5 confocal microscope with a 63× oil objective. Intensity measurements were quantified in a fixed region of interest surrounding the centrosome using Volocity software and images were processed in Adobe Photoshop 4.0.

### Data Availability.

Sequence data that support the findings of this study have been deposited in GenBank with the accession code CP029823. The structural coordinate has been deposited in the Protein Data Bank under accession code 6OQA. All other relevant data are available from the corresponding authors. More details are described in *SI Appendix*, *SI Materials and Methods*.

## Supplementary Material

Supplementary File
