# Supplementary material for: Genomic discovery of an evolutionarily programmed modality for small-molecule targeting of an intractable protein surface
Source: Proc Natl Acad Sci U S A. 2020 Jun 30;117(29):17195–203. doi: 10.1073/pnas.2006560117 (PMC7382241; doi:10.1073/pnas.2006560117)
Supplement: Supplementary File [file pnas.2006560117.sapp.pdf]

PNAS
www.pnas.org

Supplementary Information for

Genomic discovery of an evolutionary programmed modality for small-molecule targeting of an intractable protein surface

Uddhav K. Shigdel[1,a*], Seung-Joo Lee[1b*], Mathew E. Sowa[1,c*], Brian R. Bowman[1,d*], Keith Robison[1,e], Minyun Zhou[1,f], Khian Hong Pua[1,g], Dylan T. Stiles[1,e], Joshua A. V. Blodgett[1,h], Daniel W. Udwary[1,i], Andrew T. Rajczewski[1,j], Alan S. Mann[1,k], Siavash Mostafavi[1,l], Tara Hardy[2], Sukrat Arya[2,m], Zhigang Weng[1,n], Michelle Stewart[1,o], Kyle Kenyon[1,e], Jay P. Morgenstern[1,e], Ende Pan[1,p], Daniel C. Gray[1,e], Roy M. Pollock[1,c], Andrew M. Fry[2], Richard D. Klausner[3]§, Sharon A. Townson[1,q], Gregory L. Verdine[1,4,5,a,r]§

[1]Warp Drive Bio, Inc., a subsidiary of Revolution Medicines, Inc., 700 Saginaw Drive, Redwood City, CA 94063, USA

[2]Department of Molecular and Cell Biology, University of Leicester, Leicester LE1 7RH, UK

[3]Lyell Immunopharma, 400 E Jamie Court, Suite 301, South San Francisco, CA 94080

[4]Department of Stem Cell and Regenerative Biology, Harvard University, Cambridge, MA 02138, USA.

www.pnas.org/cgi/doi/10.1073/pnas.2006560117

[5]Department of Stem Cell and Regenerative Biology, Chemistry and Chemical Biology, and Molecular and Cellular Biology Harvard University, Cambridge, MA 02138, USA.

Present addresses:

[a]LifeMine Therapeutics, 100 Acorn Park Drive, Cambridge, MA 02140, USA

[b]Beam Therapeutics, 26 Landsdowne Street, Cambridge, MA 02139, USA

[c]C4 Therapeutics, Inc., 490 Arsenal Way, Suite 200, Watertown, MA 02472, USA

[d]Inzen Therapeutics, 1 Kendall Square, Cambridge, MA 02143, USA

[e]Ginkgo Bioworks, 27 Drydock Avenue, Boston, MA 02210, USA

[f]Kronos Bio, 21 Erie Street, Suite H, Cambridge, MA 02139, USA

[g]**p53 Laboratory (p53Lab),** Agency for Science, Technology, and Research (A*STAR), Singapore 138648, Singapore

[h]Department of Biology, Washington University in St. Louis, St. Louis, MO 63130, USA

[i] DOE Joint Genome Institute, Lawrence Berkeley Labs, 2800 Mitchell Drive, Walnut Creek, CA 94598, USA

[j]Department of Biochemistry, Molecular Biology, and Biophysics, University of Minnesota, Minneapolis, MN 55455, USA

[k]Agios Pharmaceuticals, 88 Sidney Street, Cambridge MA, 02139, USA

[l]Morphic Therapeutic, 35 Gatehouse Drive, Waltham, MA 02451, USA

[m]University of Oxford, Nuffield Department of Clinical Neurosciences, Oxford OX3 9DU, UK

[n]Blueprint Medicines, 215 1st Street, Cambridge, MA 02142, USA

[o]Bristol-Myers Squibb, 100 Binney St, Cambridge, MA 02142, USA

[p]Amgen 360 Binney Street, Cambridge, MA 02141

[q]Kymera Therapeutics, 300 Technology Square, Cambridge, MA 02139, USA

[r]FogPharma, 100 Acorn Park Drive, Cambridge, MA 02140, USA

* These authors contributed equally to this work

§ Corresponding authors

Email: G.L.V. (gregory_verdine@harvard.edu)
Email: R.D.K. (klausner.rick@gmail.com)

This PDF file includes:

SI Materials and Methods

SI Data

Figures S1 to S10

Tables S1 to S5

SI References

Materials and Methods

Discovery of X1 in Genomic Database

DNA for approximately 135,000 actinomycete strains were obtained from partner organizations, representing legacy pharmaceutical collections or publicly available strains. DNA was pooled at a depth between 40 and 480 strains per pool by mixing equal volumes of each sample. Pools were sequenced by WuXi Genomics (Shanghai China) using Illumina HiSeq 2x100 chemistry. FASTQ files were assembled with Ray[43] with the kmer length set to 41. BLASTX was used to search the assembled contigs against all known KCDA sequences.

Sequences positive for KCDA were used to design PCR primers in the TaqMan format, which was used to screen 1:10 dilutions of the individual input DNAs. Samples with TaqMan Ct values of 25 or less were considered positives and sequenced individually on MiSeq using 2x125, 2x250 or 2x300 chemistry and assembled with Ray as before or with SPAdes[44]. Confirmation that a strain contained a large PKS cluster next to the KCDA, as well as other homologues of rapamycin cluster genes, resulted in acquisition of the strain, growth and isolation of high molecular weight DNA by phenol-chloroform extraction. 50-100X coverage was generated using Pacific Biosciences SMRT technology and assembled with HGAP (Chin).

Strain Engineering and Fermentation

See Table S4 and S5 for the lists of strains and plasmids used in this study, and for product titer information. Strain-level engineering (*rpsL* mutant) and cluster-level engineering (+LAL and Δ*cypB*) yielded an 8.25-fold increase in WDB002 titer.

Bacterial Strains, Culture Conditions, and Molecular Methods.

E. coli was grown in Brain-heart infusion or Lennox broth with apramycin 50 ug/ml. *S. malaysiensis* was routinely grown on ISP2 (Becton, Dickinson & Co, Franklin Lakes, NJ), supplemented with antibiotics as needed. ISP4 medium (Becton, Dickinson & Co, Franklin Lakes, NJ) was used for sporulation. Liquid cultures of *Streptomyces* were grown in GYM.

Spontaneous streptomycin resistant colonies were isolated by plating spores of S22 on ISP2 with streptomycin at 20 ug/ml and incubated for 5-7 days at 30°C. Resistant colonies were restreaked to ISP2 with streptomycin 20 ug/ml and amplified by PCR using primers S22 rpsL Forward and S22 rpsL Reverse. Point mutations were

confirmed by Sanger sequencing (Genewiz, South Plainfield, NJ) using the same primers. S49 was sporulated and used for generation of the knock out strains.

Fermentation was carried out by picking colonies and inoculating to 20 ml GYM plus apramycin 50 ug/ml in a 125-ml baffled flask and cultured at 30°C for 48 hours. Cultures were homogenized with a glass tissue homogenizer and 10% (v/v) was transferred to a secondary seed culture of GYM and incubated for 24 hours at 30°C. A 5% (v/v) inoculum was added to 500 ml of 8430 production media [Per Liter: 10 g Proflo cottonseed meal (ADM, Chicago, IL), 20 g D-Mannitol, 1 g Yeast Extract (Difco) 100 mg KH_2PO_4, 50 mg $MgSO_4$, 20 mg $CaCl_2$-$2H_2O$, 4 ml ProFlo oil (ADM), 2.0 ml R2 trace elements[45], pH adjusted to 6.5 with NaOH] in 2.8L Fernbachs and grown at 30°C for 6 days. Samples were harvested and processed for chemical detection.

Gene deletion constructs were created utilizing a *rpsL* dominance based counterselection strategy[46]. Marker-less deletion cassettes were created by fusion of the upstream and downstream homology regions of the cytochrome P450 genes (*cypA, cypB* or *cypA*-*cyp B*) by the method of Blodgett *et al*.[47,48]. Deletion cassettes were cloned into pJVD52.1 to generate the knockout vectors pWDB057, pWDB058, and pWDB059. These constructs were introduced to S49 by intergenic conjugation from *E. coli* JV36. Isolation of knock outs was done by the method of Blodgett *et al*.[48] Confirmed knock out strains were sporulated and pWDB041 was introduced to generate the final strains for fermentation.

A TTA-less version of the LAL gene from S18 was synthesized by Genscript (Piscataway, NJ) with the *ermE** promoter and fd terminator and cloned into pSET152 to generate pWDB041.

Conjugations were done using the method of Blodgett *et al*.[48] Briefly *Streptomyces* spores were heat treated at 50°C for 10 minutes prior to use. Overnight cultures of the donor were washed in an equal volume of Lennox broth and 2 ul added to

100 ul heat treated spores and spotted to R2 medium[44] without yeast extract or sucrose. Plates were incubated at 30°C for 20 hours for the knock outs or 37°C for 20 hours for integration, and then overlaid with 1 ml water with 2.4 mg nalidixic acid and 1.5 mg apramycin and incubated at 30°C for 3-5 days. Exconjugants were picked and restreaked to ISP2 plates with 25 ug/ml nalidixic acid and 50 ug/ml apramycin. Single colonies were picked and grown in GYM with apramycin and saved in 15% glycerol at -80°C.

Initial analysis was performed by plating 200 ul of mycelium grown in GYM for 48 hours spread to confluence on 8430 agar plates and grown for 7 days at 30°C. Extraction was done by methanol soak overnight and purified over a Strata C-18U SPE column (Phenomenex, Torrance, CA). Samples were dried under vacuum and resuspended in DMSO. Determination of production was via LC/MS.

Extraction, Isolation and Structure Determination of WDB001, WDB002, and WDB003

Large-scale pellets were extracted with EtOAc/MeOH (90/10) then filtered over celite. The combined filtrates are dried down to crude extract. The samples were first fractioned by MPLC silica gel column chromatography (25~40 uM with hexane-EtOAc as mobile phase). WDB001, WDB002, and WDB003 were then purified from enriched fractions by reversed phase HPLC chromatography (XBridge C18 column, MeOH/H2O, 0.1 % formic acid). For this study, a total of ~50 g WDB002 was isolated, 40.3 g of which was obtained via a single 500 L fermenter run. The structures of compounds were determined by the analysis of their mass spectrometry and 1D, 2D NMR data (Supplementary Information).

Chemicals

We purchased rapamycin from LC Laboratories (cat# R-5000).

Synthesis of WDB011 [(2S)-1-((4R,7S)-7-((2R,3S,4R,11S,12R)-12-benzyl-3,11-dihydroxy-4-methyltetradecan-2-yl)-2-hydroxy-4-methyl-3-oxooxepane-2-carbonyl)piperidine-2-carboxylic acid C-11 lactone]

Synthesis of WDB011 was performed using the 7-membered oxepane form of WDB002 (hereafter referred to as WDB002b). To a mixture of WDB002b [(2S)-1-((4R,7S)-7-((2R,3S,4R,6E,9E,11R,12R)-12-benzyl-3,11-dihydroxy-4-methyltetradeca-6,9-dien-2-yl)-2-hydroxy-4-methyl-3-oxooxepane-2-carbonyl)piperidine-2-carboxylic acid C-11 lactone, 5 mg, 8.2 μmol], 10% palladium on carbon (2 mg), and a stirrer bead under nitrogen was added ethyl acetate (1 mL). The flask was charged with hydrogen and stirred vigorously for 1.5 hr. The atmosphere of hydrogen was replaced with nitrogen and the reaction filtered through celite. The celite pad was washed with more ethyl acetate and the solvent evaporated in vacuo. The residue was purified by chromatography on silica gel, gradient elution ethyl acetate: hexanes 40:60 to 100:0 to afford the title compound. When this synthesis was performed using the 7-membered oxepane form (WDB002b), the corresponding 7-membered form of the product will be isolated (WDB011b). While this material is stable in the solid state and some aprotic organic solvents, in aqueous buffer an isomerization reaction occurs to provide a 6-membered pyran isomer (WDB011). Spectral data for the 7-membered ring form can be found in Supplement Data.

Protein expression and purification

We expressed human FKBP12, $CEP250_{11.4}$ and $CEP250_{29.2}$ containing an N-terminal His-tags in *E. coli* BL21(DE3)pLysS cells using standard protocols. We harvested cells by centrifugation and resuspended the pellets with 500 mM NaCl and 3 mM TCEP in 1X

PBS buffer (Lysis buffer). We lysed the cells by sonication, and clarified the lysate by centrifugation at 23,000g for 20 min. We added the clarified lysate to Ni-NTA beads (QIAGEN), equilibrated them with the lysis buffer, and eluted the protein in 1X PBS; 250 mM imidazole; 3 mM TCEP. We removed the N-terminal His-tags with TEV protease (Sigma), followed by size exclusion chromatography using Superdex 75 (GE Healthcare) in 12.5 mM HEPES pH 7.4; 75 mM NaCl. We also expressed a fragment of human CEP250, consisting of residues 2134-2231, with an N-terminal GST-His-tag; we harvested, resuspended and lysed this protein as described above. We first purified $CEP250_{11.4}$ protein by Glutathione Sepharose 4B (GE Healthcare) and further purified it by size exclusion chromatography using Superdex 200 (GE Healthcare). We expressed and purified His-FKBP12, AVI-tagged FKBP12, HA-FKBP12 from *E. coli* BL21(DE3) pLysS cells.

Single-point mutations on $CEP250_{29.2}$ (Q2191A, V2193L, A2194T, and L2197A) were introduced by QuikChange II site-directed mutagenesis kit (Agilent), and the proteins were expressed and purified as described for WT-$CEP250_{29.2}$. Human HSBP1 (1-76) containing an N-terminal GST tag was expressed in *E. coli* BL21(DE3)pLysS cells using standard protocols. Lysed cells were purified by Glutathione affinity chromatography in PBS buffer using Glutathione Sepharose 4B beads (GE Healthcare). We removed the N-terminal GST-tag with TEV protease (Sigma), followed by purification on a Resource Q column (GE Healthcare) in HEPES-NaCl pH 7.5 buffer.

Identification of FKBP12-interacting proteins

We performed AP-MS/MS experiments using HEK293T cell lysates in a buffer containing 50 mM HEPES, pH 7.5; 120 mM NaCl; 0.5% octyl-beta-glucoside; 2 mM $MgCl_2$, 2 mM $CaCl_2$ and a protease inhibitor cocktail (Roche). We resuspended the cell pellets in lysis buffer, gently sonicated and centrifuged them; we used the supernatant

from this for all subsequent experiments. We added N-terminal AVI-tagged and biotinylated FKBP12 to the cleared lysate to a final concentration of 4 μM and supplemented it either with DMSO or 10 μM compound and 60 μL of lysis buffer-equilibrated, 50% slurry of Streptavidin agarose (Pierce). We allowed the reaction to proceed at 4°C with gentle rocking for 60 min, after which time we washed the samples 3X with lysis buffer and 3X with lysis buffer without detergent. We eluted the remaining proteins 50 mM HEPES, pH 8.5; 7 M urea at room temperature for 2 x 15 min. Due to the strong nature of the biotin-streptavidin interaction, nearly all biotin-FKBP12 was retained on the resin. We digested the eluted samples in 4 M urea with Lys-C for 2 hrs at 37°C, and then diluted them in 50 mM HEPES, pH 8.5; 0.9 M urea and added trypsin for digestion overnight at 37°C. After digestion, we desalted the peptides using C18 spin columns and analyzed them using LC-MS/MS on a Velos-Pro OrbiTrap mass spectrometer operated in data-dependent Top20 mode with CID-based fragmentation. We identified the peptides using Sequest with a target-decoy database[49].

Identification of CEP250 binding domain

We initially homed in on the minimal CEP250 domain responsible for engagement with FKBP12-WDB002 using truncated forms of the protein based on a combination of those from Mayor et al.[34] and using secondary structure prediction software (JPred). These truncated proteins included the N-terminal domain (amino acids 1-362), two intermediate domain constructs (I1: 380-1200 and I2: 1219-2124) and the C-terminal domain (1982-2442). We expressed these constructs in *E. coli* with N-terminal 6xHis tags and purified them using Ni2+-NTA agarose resin followed by size exclusion chromatography over a Sephadex 200 column. Only the C-terminal domain made WDB002-dependent interactions with FKBP12. To further reduce the binding region in the C-terminal domain, we generated successive truncations from both the N and C termini at 30 amino acid

intervals. We cloned these deletion constructs into an *in vitro* transcription/translation vector (pT7CFE1-NHis) and then expressed them using HeLa cell extracts (Pierce #88892).

We screened each deletion construct for the ability to engage HA-tagged FKBP12 in the presence of WDB001. We incubated HA-FKBP12 with each His-tagged CEP250 fragments along with either DMSO or WDB001 at 4°C for 1 hr. We enriched for the HA-FKBP12-associated proteins using anti-HA agarose and used western blots with anti-His antibodies to identify the interacting CEP250 fragments. We selected the protein spanning amino acids 1981-2281 as a minimized binding region since it was the smallest fragment that displayed WDB001-dependent FKBP12 binding. We considered any CEP250 fragments that we identified in both DMSO and WDB001 samples mis-folded so we eliminated them.

Pull-down assay to assess binding of $CEP250_{29.2}$ to WDB compounds

We incubated 20 µL of streptavidin magnetic beads (Pierce) with 0.5 µM biotinylated-FKBP12, 2.5 µM $CEP250_{29.2}$ and 5 µM of compound or volume-matched DMSO in binding buffer (10 mM HEPES, pH 7.4; 150 mM NaCl; 1 mM 2-mercaptoethanol; 0.005% (v/v) Surfactant-P20; 1% BSA) and rotated the reaction overnight at 4 °C. We harvested the beads with a magnet and washed them four times with wash buffer (10mM HEPES, pH 7.4; 150 mM NaCl, 1 mM 2-mercaptoethanol; 0.005% (v/v) Surfactant-P20). We eluted the proteins from the beads in SDS loading buffer by heating for 5 min. We collected and analyzed the eluate by SDS-PAGE and visualized with Coomassie blue staining.

Ternary complex formation and crystallization

We incubated FKBP12 with three-molar excess of X1-encoded compounds at 4°C overnight. We then added three-molar excess of this binary complex to $CEP250_{29.2}$ or $CEP250_{11.4}$ and incubated it at 4°C overnight. We isolated ternary complexes from unbound proteins and compounds by size exclusion chromatography using Superdex 200 in 12.5 mM HEPES pH 7.4; 75 mM NaCl. We obtained WDB002-ternary complex crystals by mixing 0.2 μl of isolated complex at 15 mg/ml with 0.2 μl of the reservoir solution containing 0.1 M HEPES 7.0; 0.2 M sodium malonate; 21% PEG 3350 by the sitting-drop vapor diffusion method at 20-22°C. We transferred the crystals to cryoprotectant solution containing an additional 15-20% glycerol before flash-freezing in liquid nitrogen prior to data collection.

Data collection and structure determination

We collected the WDB002 ternary complex dataset at the 21ID-G (LS-CAT) beamline of the Advanced Photon Source and used HKL program suites to process the datasets. We obtained the initial molecular-replacement solution for the WDB002-ternary complex by PHASER in the CCP4 suite, using the coordinates of previously determined FKBP12 structure (PDB 1FKD) but omitting CEP250 as search models. The model was built through iterative cycles of manual model building of CEP250 in COOT[50] and structure refinement using REFMAC5[51]. The Ramachandran plots, calculated by MolProbity[52], contain no residues in disallowed regions. We prepared all structure model figures with PyMol (The PyMOL Molecular Graphics System, Version 1.3, Schrödinger). For details on the data collection and structure refinement, see Table S3.

Surface Plasmon Resonance (SPR)

We analyzed small-molecule binding kinetics at 25°C on a Biacore S200 SPR instrument (GE Healthcare) in running buffer (10 mM HEPES, pH 7.4; 150 mM NaCl; 0.05% (v/v)

Surfactant-P20; 2% DMSO) that we prepared and filtered before use. To assess binding kinetics of FKBP12 binding ligands, we immobilized biotinlayted-FKBP12 on a CAP sensor chip to afford a highly stable and active surface suitable for kinetic measurements. We primed and charged the sensor chip with SA first. We passed 10 ug/ml of biotinlayted-FKBP12 over the sensor surface at a flow rate 2 µl/min for 60 s to stably immobilize ~600 RU of biotinlayted-FKBP12. We injected the compounds over a concentration range over target and control flow cells with multi-cycle runs at a flow rate of 50 µl/min for 100 seconds. After 300 seconds of dissociation, we regenerated the sensor surface with regeneration solution provided in the CAP chip Kit (GE Healthcare). We performed ternary binding kinetic evaluations of HSBP1, $CEP250_{11.4}$, WT $CEP250_{29.2}$, and mutants of $CEP250_{29.2}$ for each compound using a similar capture protocol except that a final of 100 RU of biotinlayted-FKBP12 was captured on the CAP sensor chip surface. After the surface was prepared, we used an ABA injection mode to inject compounds at a flow rate of 30 µl/min for 120 seconds to saturated FKBP12 on the chip surface, which allows us to isolate the binding of target proteins. To measure association, we injected the CEP250 or HSBP1 proteins over a concentration range in the presence of compound at a flow rate of 30 µl/min for 200 seconds. Dissociation was measured in the presence of compounds for 290 second. We obtained binding curves and kinetic data after subtracting the blank values, and we analyzed the data by fitting it to a 1:1 Langmuir-binding model provided by the Biacore S200 evaluation software.

Cell lines and reagents

The full length human FKBP12 ORF was cloned into the retroviral vector pMSCV-N-Flag-HA-IRES-PURO to create pMSCV-N-Flag-HA-FKBP12-IRES-PURO, a retroviral vector capable of expressing an amino terminal FLAG and HA tagged full length FKBP12 protein. U2OS:FLAG-FKBP12 cells were generated by puromycin selection

following stable transduction with virus generated by packaging this vector in 293T cells. U2OS:FLAG-FKBP12 cells were then maintained in Dulbecco's modified Eagle's medium (DMEM) supplemented with 10% fetal calf serum, 100 IU/ml penicillin and 100 µg/ml streptomycin at 37°C and 5% CO_2. Transfections were performed with Fugene HD (Roche) according to the manufacturer's instructions. Cells were incubated with WDB002, WDB003 or FK506 at 10 µM for 24 hours, unless otherwise indicated, and stimulated with EGF (Source Bioscience UK; 100 ng/µl) for 3 hours where indicated.

Immunofluorescence microscopy

U2OS: FLAG-FKBP12 cells were fixed with ice-cold methanol for 30 minutes at −20°C, and immunofluorescence microscopy performed as previously described[53]. Primary antibodies were against FLAG (1:1000; Sigma F3165), CEP250 (1:500; Cambridge Bioscience 14498) and pCEP250 (0.75 µg/ml 'LLEK'; ref. 36). Secondary antibodies used were Alexa-Fluor-488-conjugated and Alexa-Fluor-594-conjugated goat anti-mouse-IgG and goat anti-rabbit-IgGs (10 µg/ml, Invitrogen A11001 and A11012, respectively). Images were captured using a Leica TCS SP5 confocal microscope with a 63x oil objective. Intensity measurements were quantified in a fixed region of interest surrounding the centrosome using Volocity software and images processed in Adobe Photoshop 4.0.

Western blotting and immunoprecipitation

For Western blots, U2OS cells were grown as above with the addition of WDB002 (10 µM) for the times indicated. Cells lysates prepared using RIPA buffer were separated by SDS-PAGE and transferred to nitrocellulose before incubation with CEP250 and α-tubulin primary antibodies and detection using HRP-conjugated secondary antibodies and enhanced chemiluminescence. For immunoprecipitation, HeLa and U2OS stable

cell lines expressing FLAG-tagged FKBP12 were grown as above and transfected with myc-CEP250-CTD constructs using HD Fugene. After 24 hours, cells were lysed using RIPA buffer and lysates incubated with FLAG-resin (M2 antibody; Sigma A2220). Immunoprecipitates were analysed by Western blotting with FLAG (Sigma F3165) and myc (Cell Signalling 2276) antibodies.

Statistical analysis

Histograms and dot plots were prepared using Graphpad Prism 7.00. Experiments (n=3, at least 25 cells counted per experiment) were collated and statistics done using either a paired t-test or 1 way ANOVA followed by post hoc testing (Dunnetts multiple comparison test).

SI Data

WDB001

Chemical Formula: $C_{36}H_{53}NO_6$

Molecular weight: 595.4

^{1}H NMR (600 MHz, $CDCl_3$)

δ 7.29 (t, J=7.6 Hz, 2H), 7.21 (t, J=7.3Hz, 1H), 7.16 (d, J=7.3Hz, 2H), 5.69 (m, 1H), 5.52 (m, 1H), 5.42 (m, 1H), 5.35 (m, 1H), 5.31 (m, 1H), 4.61 (d, J=13.9, 1H), 4.42 (m, 1H), 3.77 (m, 1H), 3.55 (dd, J=10.4, 2.8 Hz, 1H), 2.80-2.70 (m, 4H), 2.65-2.55 (m, 3H), 2.17 (m, 1H), 2.11 (m, 1H), 1.99 (m, 1H), 1.86 (m, 1H),1.80-1.20 (m, 13H), 1.07 (m, 1H), 1.06 (d, J=6.7, 3H), 0.96 (d, J=6.7, 3H), 0.91 (t, J=7.4, 3H), 0.77 (d, J=6.8 Hz, 3H)

^{13}C NMR (125 MHz, $CDCl_3$)

δ 172.4, 168.9, 140.5, 132.1, 128.9, 128.8, 128.4, 127.3, 126.4, 126.0, 98.2, 77.4, 71.1, 70.3, 55.0, 45.8, 39.3, 39.2, 39.1, 38.9, 36.1, 35.3, 34.2, 33.4, 30.2, 28.2, 27.7, 24.6, 22.4, 20.3, 17.1, 16.7, 11.7, 9.4.

WDB002

Chemical Formula: $C_{36}H_{51}NO_7$

Molecular weight: 609.4

^{1}H NMR (600 MHz, $CDCl_3$)

δ 7.28 (t, J=7.6 Hz, 2H), 7.19 (t, J=7.3, 1H), 7.12 (d, J=7.3, 2H), 5.45-5.35 (m, 2H), 5.53 (m, 2H), 5.30 (s, 1H), 5.26 (m, 1H), 3.87 (m, 1H), 3.55-3.45 (m, 2H), 3.05 (m, 1H), 2.85-2.70 (m, 3H), 2.50 (m, 1H), 2.40-2.25 (m, 2H), 2.20 (m, 1H), 2.05 (m, 1H), 1.85-1,27 (m,

14H), 1.01 (d, J=6.7, 3H), 0.97 (d, J=6.5 Hz, 3H), 0.87 (t, J=7.4 Hz, 3H), 0.86 (d, J=6.7 Hz, 3H)

^{13}C NMR (125 MHz, $CDCl_3$)

δ 196.9, 169.6, 165.4, 140.3, 133.9, 129.1, 128.9, 128.3, 127.9, 126.9, 126.0, 99.0, 77.6, 73.5, 73.0, 51.9, 45.4, 44.6, 40.0, 36.0, 35.4, 34.9, 34.6, 34.2, 29.6, 27.6, 25.9, 25.0, 22.7, 21.2, 16.8, 16.2, 10.8, 10.7.

WDB003

$C_{36}H_{49}NO_8$

Molecular weight: 623.3

^{1}H NMR (500 MHz, Benzene-d_6)

δ 7.30 (m, 1H), 7.20-7.10 (m, 6H), 7.10-7.06 (m, 3H), 6.99 (m, 2H), 5.65-5.55 (m, 2H), 5.45 (m, 1H), 5.25-5.15 (m, 2H), 4.98 (dd, J=15.5, 7.3 Hz, 1H), 4.89 (dd, J=8.9, 5.0 Hz, 1H), 4.67 (dd, J=15.3, 8.8 Hz, 1H), 4.45 (m, 2H), 4.20 (m, 1H), 4.13 (m, 1H), 3.87 (m, 1H), 3.56 (m, 2H), 3.35-3.05 (m, 3H), 2.71 (m, 2H), 2.65-2.50 (m, 2H), 2.50-2.30 (m, 4H), 2.08 (m, 1H), 1.92 (m, 1H), 1.80-1.45 (m, 16H), 1.71 (d, J=6.8 Hz, 3H), 1.54 (d, J=6.8 Hz, 3H), 1.40-0.90 (m, 29H), 1.24 (d, J=6.5 Hz, 3H), 1.18 (d, J=6.6 Hz, 3H), 1.07 (d, J=6.8 Hz, 3H), 1.01 (m, J=6.7 Hz, 3H), 0.73 (t, J=7.5 Hz, 3H), 0.69 (t, J=7.5 Hz, 3H)

^{13}C NMR (126 MHz, Benzene-d_6)

δ 201.5, 199.9, 197.8, 191.6, 170.4, 169.5, 166.8, 166.6, 145.4, 144.8, 140.6, 140.5, 133.9, 131.3, 129.7, 129.4, 129.3, 128.8, 128.8, 128.4, 128.3, 126.6, 126.5, 126.0, 100.0, 99.3, 80.6, 78.2, 73.1, 72.4, 71.7, 70.6, 57.1, 52.6, 51.9, 51.1, 45.8, 45.4, 44.2,

42.5, 42.2, 39.8, 35.8, 35.7, 35.6, 34.1, 33.6, 33.4, 29.9, 29.9, 29.3, 28.2, 27.4, 27.1, 25.1, 25.1, 22.3, 22.2, 21.3, 21.2, 16.6, 16.2, 14.6, 13.7, 11.2, 11.1, 10.6, 9.5.

WDB011

$C_{36}H_{55}NO_7$

Molecular weight: 614.4

^{1}H NMR ($CDCl_3$, 500MHz): δ 7.28 (m, 2H), 7.19 (m, 1H), 7.13 (d, *J* = 6.98 Hz, 2H), 5.65 (s, 1H), 5.26 (d, *J* = 4.92 Hz, 1H), 5.11 (m, 1H), 4.67 (d, *J* = 13.02 Hz, 1H), 4.02 (dd, *J* = 10.67, 1.13 Hz, 1H), 3.35 (m, 1H), 3.23-3.10 (m, 2H), 2.72 (dd, *J* = 13.85, 5.50 Hz, 1H), 2.50 (dd, *J* = 13.93, 9.25 Hz, 1H), 2.39 (m, 1H), 1.95-1.73 (m, 5H), 1.71-1.15 (m, 25H), 1.03 (d, *J* = 6.71 Hz, 3H), 0.85 (t, *J* = 7.42 Hz, 3H), 0.79 (d, *J* = 6.82 Hz, 3H)

^{13}C NMR ($CDCl_3$, 126MHz): δ 210.5, 170.3, 167.4, 140.5, 129.0, 128.3, 126.0, 97.8, 79.1, 76.9, 71.1, 52.0, 45.9, 43.9, 43.5, 39.9, 36.3, 35.1, 33.1, 32.3, 31.9, 29.1, 27.9, 27.1, 25.8, 25.1, 23.4, 22.1, 21.1, 20.0, 17.0, 16.6, 11.5, 8.9

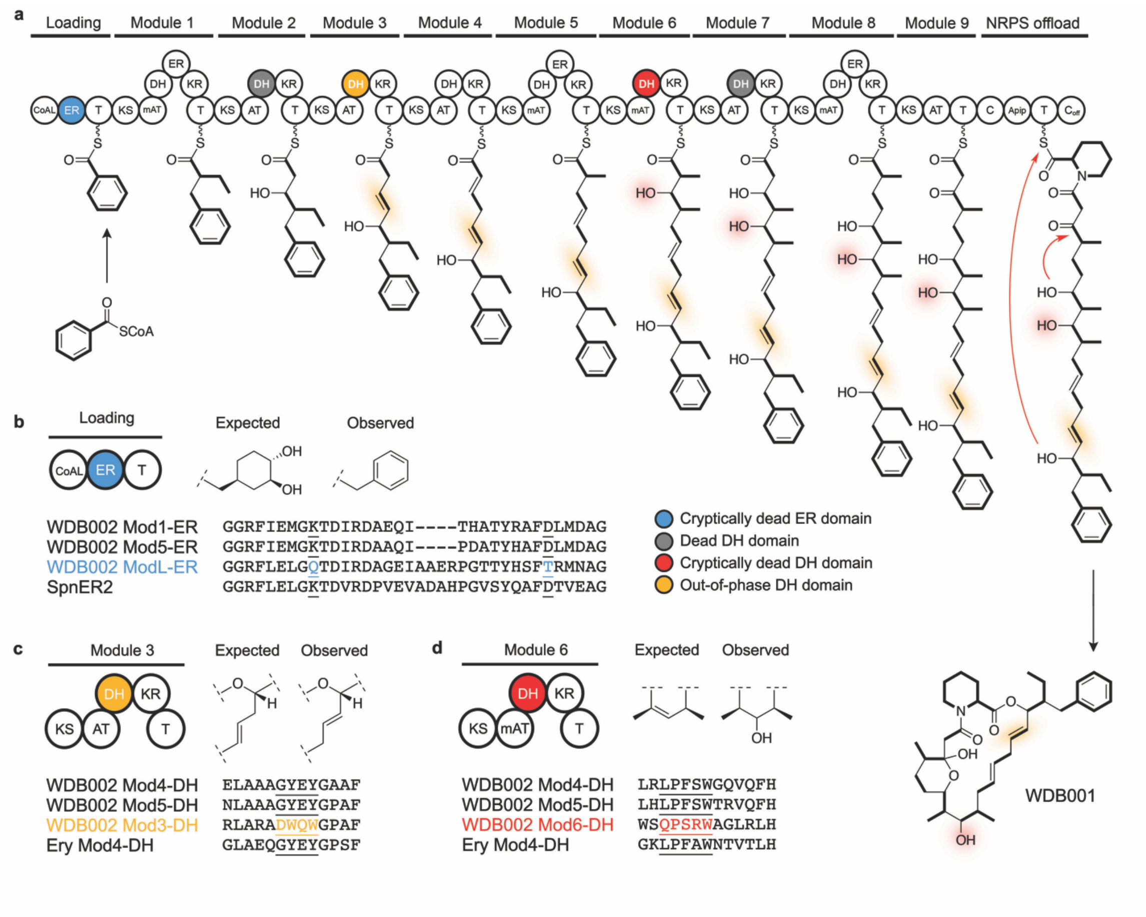

Fig. S1 | Biosynthesis of X1-encoded compound WDB001. a, Proposed assembly line biosynthesis comprising 1 loading module, 9 PKS modules and a final NRPS module responsible for loading pipecolate. DH domains are colored by function (active = white, out-of-phase = yellow and enzymatically-dead = red.) b, WDB001, WDB002 and WDB003 possess an aromatic starter unit consistent with mutations in the loading ER domain active site Lys-Asp dyad. c, C22-23 olefin is out of phase according to our prediction from the PKS modules. Compared with DH sequences from the other X1 modules, two scaffolding tyrosines at the catalytic site are replaced by tryptophans. We propose that these substitutions are sufficient to cause this DH to promote gamma-hydrogen elimination relative to the acyl carrier protein (ACP)-bound thioester instead of the normal alpha-hydrogen elimination to result in a non-canonical dehydration product. d, WDB001, WDB002 and WDB003 also possess a hydroxyl at C16 that we did not

predict based on Module 6 domain organization. Alignment of DH6 with the two active DH domains in X1, DH4 and DH5, as well as the erythromycin synthase DH4, reveals the active-site proximal residues are not conserved in X1 DH6. We postulate that these amino acid changes may compromise the ACP-docking interface, rendering the DH6 domain inactive and resulting in the hydroxyl at C16.

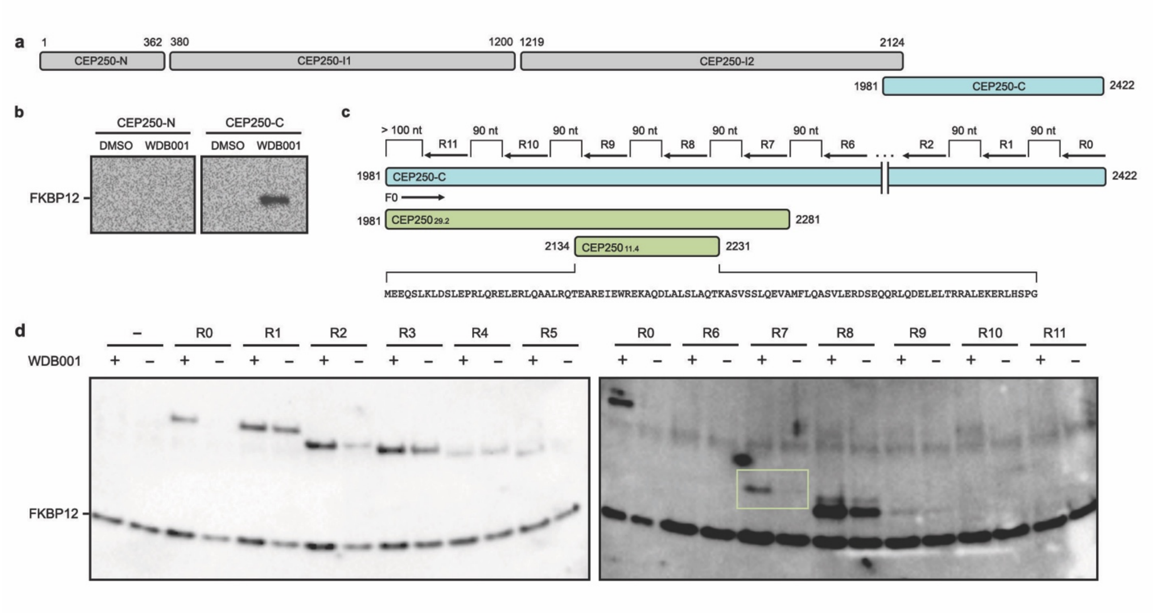

Fig. S2 | Identification of minimal CEP250 domain that binds FKBP12-WDB001. a, CEP250 was divided into four domains based on JPred secondary structure prediction. These truncated domains included the N-terminal domain (amino acids 1-362), two intermediate domain constructs (I1: 380-1200 and I2: 1219-2124) and the C-terminal domain (1982-2442). **b**, Pull-down experiments identified only the C-terminal domain of CEP250 as making WDB001-depedent interactions with FKBP12 (I1 and I2 domains not shown). **c**, CEP250 fragments with successive truncations of 30 amino acids from the C-terminal end (R0-R11) were tested for interaction with FKBP12-WDB001. **d,** Residues 1,981-2,281 (CEP250$_{29.2}$) were identified as the minimal binding region of CEP250.

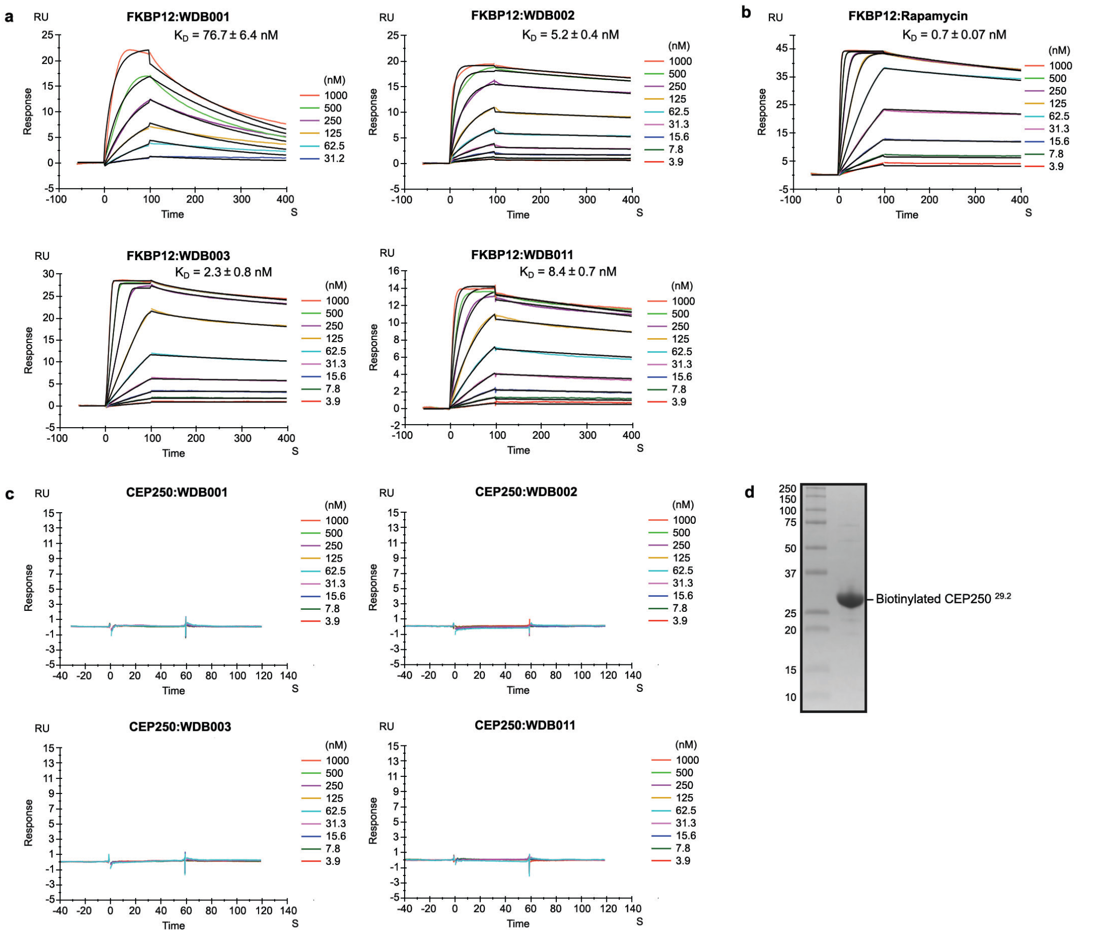

Fig. S3 | Representative SPR sensorgrams for binary K_D determination. **a**, Binary interaction of immobilized FKBP12 with X1-encoded compounds and **b** Rapamycin. **c**, Binary interaction of immobilized $CEP250_{29.2}$ with X1-encoded compounds **d**, SDS-PAGE image of the biotinylated $CEP250_{29.5}$ sample used in generating data in panel c.

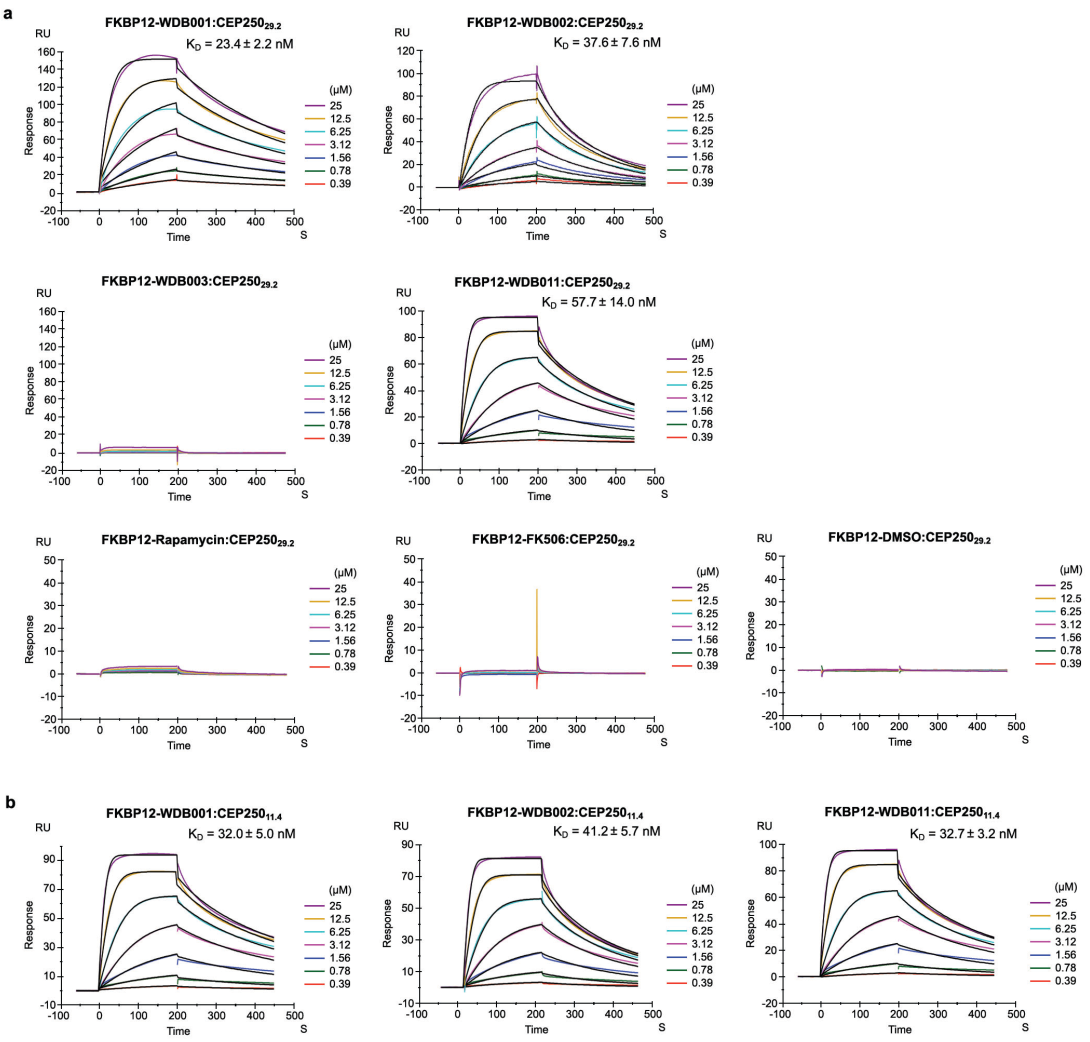

Fig. S4 | Representative SPR sensorgrams for ternary K_D determination. **a**, Ternary interaction of immobilized FKBP12-X1 binary complex with $CEP250_{29.2,}$ including Rapamycin, FK506 and DMSO controls and **b**, Ternary interaction of FKBP12-binary complexes of WDB001, WDB002 and WDB011 with $CEP250_{11.4}$.

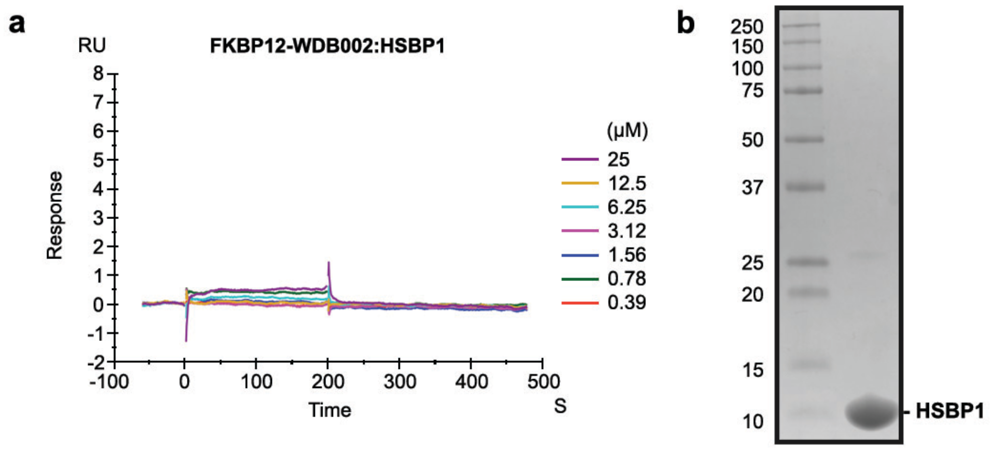

Fig. S5 | Ternary interaction of HSBP1 and FKBP12-WDB002. **a**, SPR sensorgram of ternary interaction of immobilized FKBP12-WDB002 binary complex with HSBP1 and **b**, SDS-PAGE image of purified untagged HSBP1 sample used in SPR experiments.

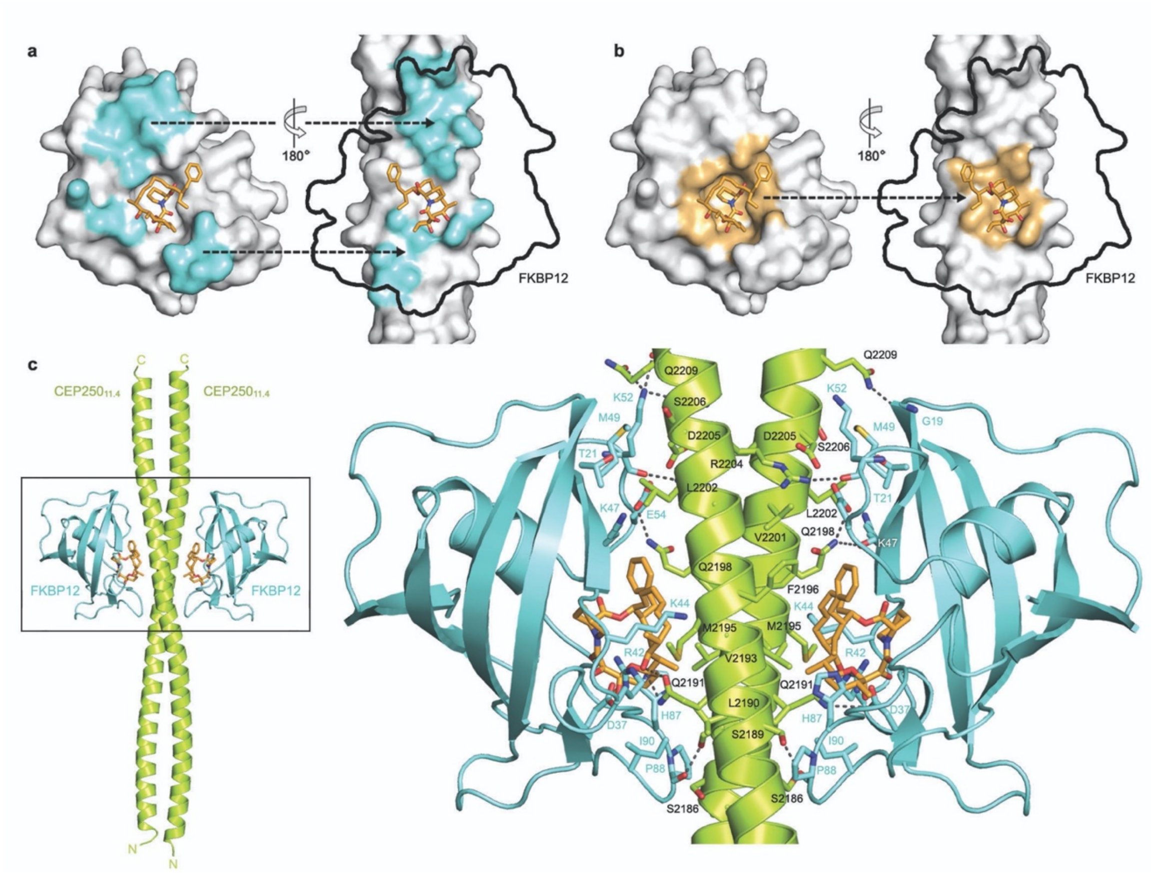

Fig. S6 | Detailed view of CEP250-WDB002-FKBP12 interface a, Contact surface of CEP250 on FKBP12-WDB002 (left) and FKBP12 on CEP250 (right, black outline). Amino acids interacting at the interface are colored cyan. **b**, Contact surface of WDB002 on FKBP12 (left) and CEP250 (right, FKBP12 contact surface as in panel a). Amino acids interacting at the interface are colored yellow. **c**, Presenter-ligand-CEP250 interface. FKBP12 (cyan), WDB002 (orange sticks) and CEP250 (green). Hydrogen bonds are depicted as dotted lines. Interface amino acids are labeled with black (CEP250) or cyan (FKBP12) text.

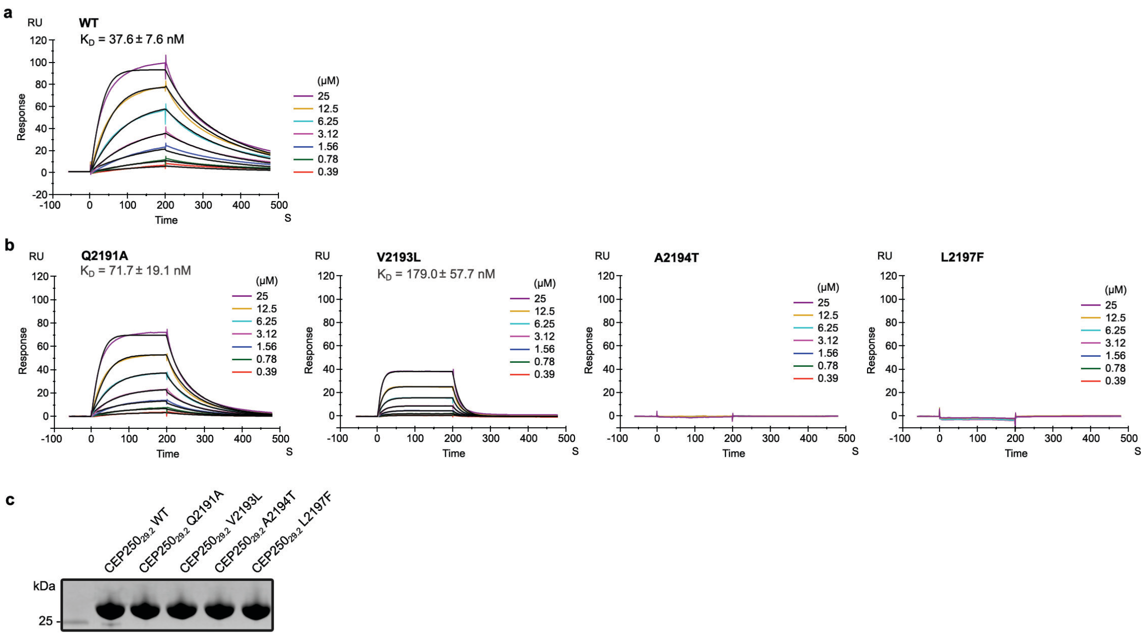

Fig. S7 | SPR sensorgrams for ternary K_D determination of CEP250 mutants a, Ternary interaction of immobilized FKBP12-WDB002 binary complex with WT $CEP250_{29.2}$, b, Ternary interaction of FKBP12-WDB002 binary complex with mutants of $CEP250_{29.2}$ and c, SDS-PAGE gel image of $CEP250_{29.2}$ protein reagents used to generate data in panels a and b.

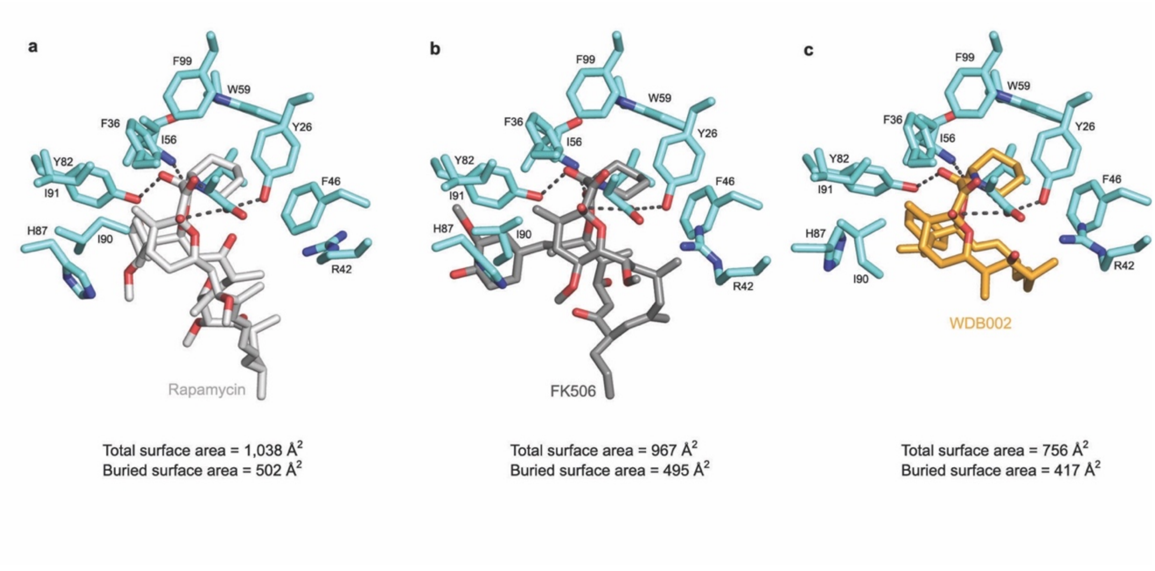

Fig. S8 | FKBP12-ligand binding comparison for Rapamycin, FK506, and WDB002

(a) Rapamycin bound to FKBP12 in FKBP12-Rapamycin-mTOR ternary complex (PDB ID 1FAP). FKBP12 residues that compose the ligand binding and within 4.5 A from the ligand are shown in cyan sticks. Conserved hydrogen bonds between FKBP12 and the ligand is displayed as dotted lines. (b) FK506 bound to FKBP12 in FKBP12-FK506-Calcineurin complex (PDB ID 1TCO). (c) WDB002 bound to FKBP12 in FKBP12-WDB002-CEP25011.4 structure (PDB ID 6CAK). Total surface of area and buried surface area of each ligand are noted.

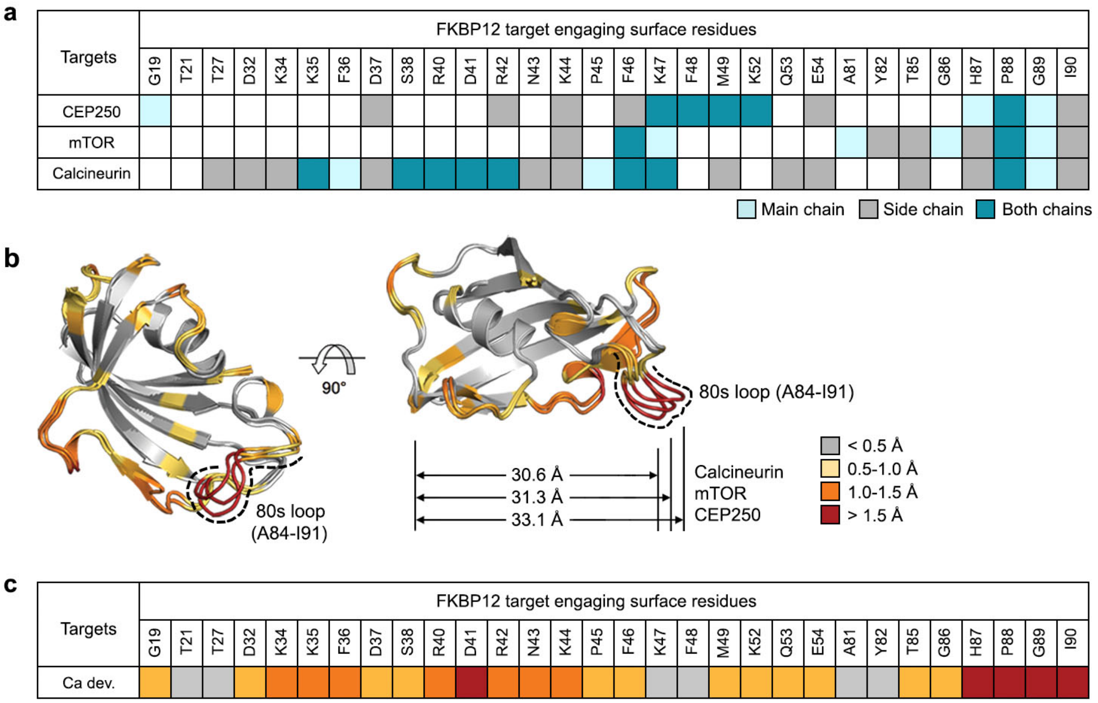

Fig. S9 | Adaptability of FKBP12 as a presenter protein. a, Graphic illustration of FKBP12 residues deployed in each complex (CEP250, mTOR, Calcineurin), indicating whether the interaction occurs via the main chain, the side chain or both. **b**, We overlayed FKBP12 in the three structures, coloring according to α-C deviation. The region of FKBP12 that binds the constant region of the ligands shows minimal deviation (grey pocket, left), whereas the target-engaging surface, including the 80s loop, varies more dramatically (colored surface at the bottom of the image, right), accounting for a 2.5 Å change in the end-to-end distance in the presenter (30.6 $Å^2$, FKBP12-FK506-Calcineurin complex; 33.1 $Å^2$, FKBP12-WDB002-CEP250 complex). **c**, Graphic illustration of α-C deviations following the same color scheme in panel b, illustrating variablility in the deployment of 80s-loop residues in the three complexes.

a

FKBP isoforms	Target-engaging residues in rapamycin complex										
	44	46	47	81	82	85	86	87	88	89	90
FKBP12	K	F	K	A	Y	T	G	H	P	G	I
FKBP12.6	K	F	K	A	Y	T	G	H	P	G	V
FKBP13	Q	F	Q	G	Y	R	G	A	P	P	K
FKBP25	K	L	K	A	Y	K	G	Q	P	A	K
FKBP51	E	F	E	A	Y	A	G	S	L	P	K
FKBP52	D	F	D	A	Y	A	G	S	P	P	K

b

FKBP isoforms	Target-engaging residues in WDB002 complex													
	19	37	42	44	46	47	48	49	52	54	87	88	89	90
FKBP12	G	D	R	K	F	K	F	M	K	E	H	P	G	I
FKBP12.6	G	D	R	K	F	K	F	R	K	E	H	P	G	V

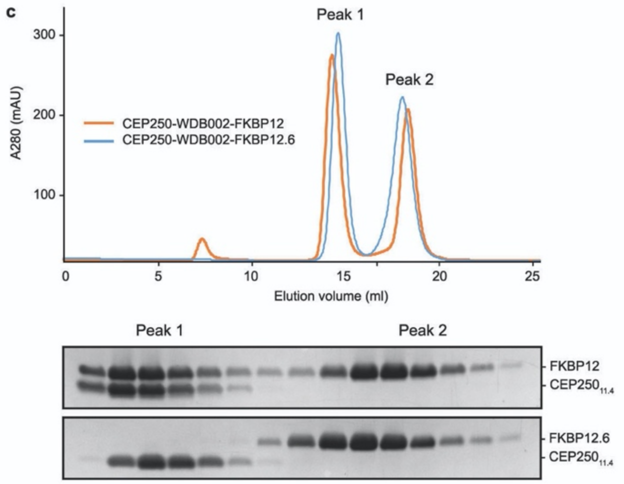

Fig. S10 | Comparison of FKBP target engagment residues for WDB002 and Rapamycin. a, Rapamycin can form a ternary complex with mTOR through myriad FK506-binding proteins (FKBP12, FKBP12.6, FKBP13, FKBP25, FKBP51 and FKBP52)[31]. The interacting amino acids in the FKBP12-rapamycin-mTOR complex are not well conserved (red delineates amino acid substitutions). In the FKBP51-rapamycin-mTOR and FKBP52-rapamycin-mTOR complexes, the FK506-binding proteins deploy distinct but overlapping residues with those in the FKBP12-rapamycin-mTOR complex[31]. **b**, FKBP12 drives the FKBP12-WDB002-CEP250 interaction. FKBP12.6, contains only three amino acid changes from FKBP12 in the residues deployed at the ternary complex interface (red delineates amino acid substitutions). **c**, FKBP12.6 does not support ternary complex formation.

Table S1 | Novel and known FK gene clusters discovered via genome mining from

Actinomycetes

**Cluster**	**Count**	**Rank**	**Estimated Frequency (%)**	**Strains per cluster**	**Status**
**X1**	44	1	3.26×10^{-4}	3,068	Novel
**Rapamycin**	23	2	1.70×10^{-4}	5,870	Known
**FK506/FK520**	22	3	1.63×10^{-4}	6,136	Known
**X11**	5	4	3.70×10^{-5}	27,000	Novel
**X22**	5	4	3.70×10^{-5}	27,000	Novel
**X23**	5	4	3.70×10^{-5}	27,000	Novel
**Antascomicin**	3	7	2.22×10^{-5}	45,000	Known
**X15**	1	8	7.41×10^{-5}	135,000	Novel
**X35**	1	8	7.41×10^{-5}	135,000	Novel
**X36**	1	8	7.41×10^{-5}	135,000	Novel

Table S2 | Kinetic binding data for binary and ternary complex formation

	Binary (FKBP12)			Ternary (CEP250$_{29.2}$)			Ternary (CEP250$_{11.4}$)		
	k_a (10^4 $M^{-1}s^{-1}$)	k_d ($10^{-4}s^{-1}$)	K_D (nM)	k_a (10^5 $M^{-1}s^{-1}$)	k_d ($10^{-3}s^{-1}$)	K_D (nM)	k_a (10^5 $M^{-1}s^{-1}$)	k_d ($10^{-3}s^{-1}$)	K_D (nM)
**WDB001**	4.9 ± 0.6	37.8 ± 7.2	76.7 ± 6.4	1.5 ± 0.3	3.4 ± 0.2	23.4 ± 2.2	1.2 ± 0.6	3.7 ± 0.5	32.0 ± 5.0
**WDB002**	7.9 ± 0.6	4.1 ± 0.6	5.2 ± 0.4	1.8 ± 0.6	6.7 ± 2.7	37.6 ± 7.6	1.6 ± 0.4	6.6 ± 1.8	41.2 ± 5.7
**WDB003**	34.3 ± 1.3	7.3 ± 0.5	2.3 ± 0.8	No binding	No binding	No binding	No binding	No binding	No binding
**WDB011**	7.1 ± 1.0	5.9 ± 0.7	8.4 ± 0.7	0.9 ± 0.03	5.2 ± 1.0	57.7 ± 14.0	1.6 ± 0.4	5.2 ± 0.3	32.7 ± 3.2
**Rapamycin**	78.3 ± 7.4	5.8 ± 0.4	0.7 ± 0.07	No binding	No binding	No binding	No binding	No binding	No binding
**FK506**	NA	NA	NA	No binding	No binding	No binding	NA	NA	NA

	FKBP12-WDB002		
	k_a (10^4 $M^{-1}s^{-1}$)	k_d ($10^{-2}s^{-1}$)	K_D (nM)
**CEP250$_{29.2}$-WT**	17.8 ± 5.7	67.2 ± 27.2	37.6 ± 7.6
**CEP250$_{29.2}$-Q2191A**	32.0 ± 8.1	2.3 ± 1.0	71.7 ± 19.1
**CEP250$_{29.2}$-V2193L**	2.7 ± 0.5	4.7 ± 0.7	179.0 ± 57.7
**CEP250$_{29.2}$-V2194T**	No binding	No binding	No binding
**CEP250$_{29.2}$-L2197F**	No binding	No binding	No binding
**HSBP1**	No binding	No binding	No binding

Table S3 | Data collection and refinement statistics

	$CEP250_{11.4}$-WDB002-FKBP12
**Data collection**	
Space group	$P2_1$
Cell dimensions	
a, *b*, *c* (Å)	60.8, 65.0, 136.1
α, β, γ (°)	90, 90.5, 90
Resolution (Å)	50.0-2.22 (2.30-2.22)*
R_{sym} or R_{merge}	0.085 (0.351)
$I/\sigma I$	12.9 (4.1)
Completeness (%)	99.5 (99.9)
Redundancy	3.7 (3.7)
**Refinement**	
Resolution (Å)	136.1-2.20
No. reflections	50,564
$R_{work/}$ R_{free}	0.208/0.256
No. atoms	
Protein	6,135
Ligand/ion	457
Water	226
B-factors	
Protein	56.7
Ligand/ion	61.4
Water	51.9
R.m.s deviations	
Bond lengths (Å)	0.007
Bond angles (º)	1.165

*Highest resolution shell is shown in parenthesis.

Table S4 | List of strains

**Strain**	**Organism**	**Characteristic**	**Fold increase in titer vs. S22)**	**Reference**
S18	*Streptomyces hygroscopicus* NCIMB 40319	Rapamycin LAL	n/a	54
S22	*Streptomyces malaysiensis* DSM 41697	Wild type, native X1.2	1.00	55
S49	*Streptomyces malaysiensis*	*rpsL* K43R	Data not available	This study
S328	*Streptomyces malaysiensis*	*rpsL* K43R, *ΔcypA*	0.00	This study
S333	*Streptomyces malaysiensis*	*rpsL* K43R, *ΔcypB*	3.24	This study
S363	*Streptomyces malaysiensis*	S18 LAL	3.34	This study
S582	*Streptomyces malaysiensis*	*rpsL* K43R, *ΔcypA*, S18 LAL	Data not available	This study
S583	*Streptomyces malaysiensis*	*rpsL* K43R, *ΔcypB*, S18 LAL	8.25	This study
S584	*Streptomyces malaysiensis*	*rpsL* K43R, *ΔcypAB*, S18 LAL	Data not available	This study
S181	*E. coli* JV36	*dam-3 dcm-6 metB1 galK2 galT27 lacY1 tsx-78 supE44 thi-1 mel-1 tonA31 ΔhsdRMS-mrr::*Frt*(rK- mK-) attHK::*pJK202 *(Δori*R6K-*aadA::*Frt *bla::pir)* (Tra+, Amp^{S})	n/a	48

Table S5 | List of plasmids

Plasmids	Description	Characteristic	Reference
pWDB001	pJVD52.1	Apr^R, *oriT*, *rep*$^{pSG5(ts)}$, *rep*^{pUC}, *rpsL*$^+$ shuttle vector	47
pWDB011	pSET152	Am^R, *oriT*, *rep*^{pUC}, int$^{\varphi C31}$, Apr^R, integrating vector	56
pWDB022	pWFE1	Am^R, *oriT*, *rep*^{pUC}, *int*$^{\varphi C31}$, *ermE*p*, fd terminator, integrating vector	This study
pWDB041	pWFE1 – S18 LAL	Am^R, *oriT*, *rep*^{pUC}, *int*$^{\varphi C31}$, *ermE*p*, S18 LAL, fd terminator, integrating vector	This study
pWDB057	pJVD52.1 - S22 cypA KO	Apr^R, *oriT*, *rep*$^{pSG5(ts)}$, *rep*^{pUC}, *rpsL*$^+$ S22 cypA KO	This study
pWDB058	pJVD52.1 - S22 cypB KO	Apr^R, *oriT*, *rep*$^{pSG5(ts)}$, *rep*^{pUC}, *rpsL*$^+$ S22 cypB KO	This study
pWDB059	pJVD52.1 - S22 cypAB KO	Apr^R, *oriT*, *rep*$^{pSG5(ts)}$, *rep*^{pUC}, *rpsL*$^+$ S22 cypAB KO	This study

References

43. Boisvert, S., Laviolette, F. & Corbeil, J. Ray: simultaneous assembly of reads from a mix of high-throughput sequencing technologies. Journal of computational biology : a journal of computational molecular cell biology 17, 1519-1533, doi:10.1089/cmb.2009.0238 (2010).
44. Bankevich, A. et al. SPAdes: a new genome assembly algorithm and its applications to single-cell sequencing. Journal of computational biology : a journal of computational molecular cell biology 19, 455-477, doi:10.1089/cmb.2012.0021 (2012).
45. Hopwood, D. A. & Wright, H. M. Bacterial protoplast fusion: recombination in fused protoplasts of Streptomyces coelicolor. Molecular & general genetics : MGG 162, 307-317 (1978).
46. Hosted, T. J. & Baltz, R. H. Use of rpsL for dominance selection and gene replacement in Streptomyces roseosporus. Journal of bacteriology 179, 180-186 (1997).
47. Blodgett, J. A. V. et al. Unusual transformations in the biosynthesis of the antibiotic phosphinothricin tripeptide. Nature Chemical Biology 3, 480-485, doi:10.1038/nchembio.2007.9 (2007).
48. Blodgett, J. A. V. et al. Common biosynthetic origins for polycyclic tetramate macrolactams from phylogenetically diverse bacteria. Proceedings of the National Academy of Sciences of the United States of America 107, 11692-11697, doi:10.1073/pnas.1001513107 (2010).
49. Elias, J. E. & Gygi, S. P. Target-decoy search strategy for increased confidence in large-scale protein identifications by mass spectrometry. Nature methods 4, 207-214, doi:10.1038/nmeth1019 (2007).
50. Emsley, P., Lohkamp, B., Scott, W. G. & Cowtan, K. Features and development of Coot. Acta crystallographica. Section D, Biological crystallography 66, 486-501, doi:10.1107/S0907444910007493 (2010).

51. Vagin, A. A. et al. REFMAC5 dictionary: organization of prior chemical knowledge and guidelines for its use. Acta crystallographica. Section D, Biological crystallography 60, 2184-2195, doi:10.1107/S0907444904023510 (2004).
52. Chen, V. B. et al. MolProbity: all-atom structure validation for macromolecular crystallography. Acta crystallographica. Section D, Biological crystallography 66, 12-21, doi:10.1107/S0907444909042073 (2010).
53. Regan, L. & Fry, A. M. The Nek6 and Nek7 protein kinases are required for robust mitotic spindle formation and cytokinesis. Molecular and cellular biology 29, 3975-3990, doi:10.1128/MCB.01867-08 (2009).
54. Box, S. J. et al. 27-O-demethylrapamycin, an immunosuppressant compound produced by a new strain of Streptomyces hygroscopicus. The Journal of antibiotics 48, 1347-1349 (1995).
55. al-Tai, A., Kim, B., Kim, S. B., Manfio, G. P. & Goodfellow, M. Streptomyces malaysiensis sp. nov., a new streptomycete species with rugose, ornamented spores. International journal of systematic bacteriology 49 Pt 4, 1395-1402, doi:10.1099/00207713-49-4-1395 (1999).
56. Bierman, M. et al. Plasmid cloning vectors for the conjugal transfer of DNA from Escherichia coli to Streptomyces spp. Gene 116, 43-49 (1992).
